# Static and Dynamic Resting‐State fMRI Connectivity Show Frequency‐Resolved Associations With Intracranial EEG Connectivity

**DOI:** 10.1002/hbm.70646

**Published:** 2026-09-30

**Authors:** Tahereh Rashnavadi, Kristina Sabaroedin, William Wilson, Daniel J. Pittman, Paolo Federico, Pierre Levan

**Affiliations:** ^1^ Department of Radiology University of Calgary Calgary Canada; ^2^ Hotchkiss Brain Institute University of Calgary Calgary Canada; ^3^ Department of Paediatrics University of Calgary Calgary Canada; ^4^ Department of Clinical Neurosciences University of Calgary Calgary Canada; ^5^ Seaman Family MR Research Centre University of Calgary Calgary Canada; ^6^ Alberta Children's Hospital Research Institute University of Calgary Calgary Canada

**Keywords:** BOLD fluctuations, cross‐modal brain imaging, dynamic functional connectivity, intracranial EEG‐fMRI, neurovascular coupling, resting‐state functional connectivity, static functional connectivity

## Abstract

Resting‐state functional connectivity (FC) derived from functional MRI (fMRI) is widely used to characterize brain functional organization, yet its electrophysiological basis remains incompletely understood. Here, we investigated how electrophysiological connectivity relates to both static and dynamic fMRI connectivity using simultaneous intracranial EEG (icEEG)–fMRI recordings from 48 adults with focal epilepsy. Linear mixed‐effects models were applied to sampled electrode‐pair connections to examine frequency‐resolved associations between band‐limited icEEG connectivity and corresponding fMRI FC within non‐epileptogenic brain regions. Time‐averaged icEEG connectivity was robustly associated with spatial variation in static fMRI connectivity across sampled connections and frequency bands, with strongest effects in the beta and low‐gamma ranges. At the window level, dynamic fMRI connectivity showed significant positive associations with window‐specific icEEG FC deviations in the alpha, beta, and low‐gamma bands. Exploratory spatial stratification suggested that cross‐modal coupling varied across clinically sampled frontal, temporal, and insular region pairs, and the main findings remained robust after motion‐control analyses. Together, these findings indicate that resting‐state fMRI connectivity is associated with both average electrophysiological coupling across sampled connections and window‐level fluctuations in electrophysiological connectivity, with the strongest primary band‐wise evidence observed in the alpha, beta, and low‐gamma ranges.

## Graphical abstract

Resting‐state functional MRI (fMRI) connectivity reflects both stable electrophysiological coupling and frequency‐specific neural dynamics. Using simultaneous intracranial EEG–fMRI in 48 epilepsy patients, we show that static connectivity aligns with beta/low‐gamma coupling, whereas dynamic connectivity tracks alpha and beta fluctuations.

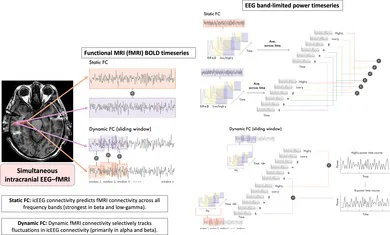

## 1 Introduction

In network neuroscience, resting‐state functional connectivity (FC) derived from functional magnetic resonance imaging (fMRI) has become a foundational tool for mapping large‐scale interactions across the human brain (Fornito and Bullmore 2010). FC is defined as the statistical dependence between spontaneous, slow (< 0.1 Hz) fluctuations in the blood oxygen level‐dependent (BOLD) signal across spatially distinct regions. Correlated low‐frequency BOLD fluctuations at rest are interpreted as reflecting coordinated neuronal activity across distributed systems and form the basis for identifying intrinsic brain networks (Liégeois and Elward 2020; Park and Friston 2013).

Conventionally, FC has been quantified over the full duration of a scan, implicitly assuming that connectivity is stationary (Biswal et al. 1995; Fox and Raichle 2007). However, extensive evidence indicates that FC is nonstationary: patterns of interregional coupling fluctuate over time even during rest, tracking changes in brain state, arousal, and consciousness (Chang and Glover 2010; Allen et al. 2014; Hutchison, Womelsdorf, Allen, et al. 2013; Preti et al. 2017). These fluctuations are commonly studied using dynamic FC approaches, including sliding‐window analyses that estimate FC across successive time windows. Dynamic FC has been shown to vary with age, cognitive ability, and clinical status (Tagliazucchi and Laufs 2014). Together, static and dynamic FC analyses have reshaped our understanding of large‐scale brain organization (Damaraju et al. 2020; Hutchison and Morton 2015; Calhoun et al. 2014; Fong et al. 2019; Smith et al. 2009). Yet despite the widespread use of fMRI FC as a marker of functional coupling, the electrophysiological processes underlying both time‐averaged and time‐varying BOLD connectivity remain incompletely understood.

Evidence linking BOLD fluctuations to neuronal activity has been derived largely from invasive electrophysiological recordings demonstrating associations between local field potential activity and BOLD signals across multiple temporal scales. Foundational non‐human primate studies showed that local field potential activity is closely related to BOLD responses, supporting an electrophysiological basis of the fMRI signal (Logothetis 2003; Logothetis and Wandell 2004). Although early work often emphasized higher‐frequency activity, particularly in the gamma range (Niessing et al. 2005), subsequent animal studies demonstrated that spontaneous BOLD fluctuations are also related to slower electrophysiological dynamics and slow modulations of local field potential power overlapping with the timescale of resting‐state BOLD fluctuations (Shmuel and Leopold 2008; Schölvinck et al. 2010). Together, these findings indicate that neural–BOLD coupling extends beyond gamma‐band activity and includes slower electrophysiological processes relevant to resting‐state fMRI.

In humans, multimodal EEG–fMRI studies have identified band‐dependent associations between electrophysiological activity and BOLD signals, including gamma–BOLD coupling and alpha‐/low‐frequency BOLD relationships (Koch et al. 2009; Mulert et al. 2010; Scheeringa et al. 2011; Tagliazucchi et al. 2012; Pan et al. 2011). However, much of this human evidence has relied on scalp EEG, which has limited spatial resolution and reduced sensitivity to deep cortical and subcortical sources, primarily reflecting synchronous activity from large populations of superficial pyramidal neurons near the cortical surface (Tao et al. 2007). In addition, high‐frequency activity recorded at the scalp is especially vulnerable to contamination by cranial and facial muscle artifacts, complicating interpretation of gamma‐band EEG‐BOLD associations (Muthukumaraswamy 2013). Simultaneous icEEG–fMRI can partly address these limitations by providing direct intracranial electrophysiological measurements during fMRI acquisition. Related EEG–fMRI and icEEG–fMRI connectivity studies further support the relevance of cross‐modal connectivity analyses in epilepsy and direct intracranial recordings (Wirsich et al. 2024; Carmichael et al. 2024), although the relative contributions of individual frequency bands remain incompletely understood.

Resting‐state fMRI FC is typically estimated from slow BOLD fluctuations in the neurovascularly relevant low‐frequency range, conventionally below 0.1 Hz. Nevertheless, even within this low‐frequency range, BOLD signals can be influenced by non‐neuronal factors, including aliased respiratory and cardiac fluctuations, scanner‐related artifacts, and in‐scanner head motion (Power et al. 2012; Birn 2012; Tong et al. 2019; Chang et al. 2009). These physiological and motion‐related confounds raise a fundamental question: to what extent does static and dynamic resting‐state fMRI FC relate to electrophysiological coupling, rather than structured non‐neuronal variance within the hemodynamic signal? Thus, although simultaneous EEG‐fMRI studies support a link between electrophysiological activity and BOLD fluctuations, they do not fully resolve how electrophysiological connectivity relates to fMRI FC across anatomically corresponding sampled connections.

Addressing this question requires direct electrophysiological recordings in humans, an opportunity afforded by intracranial EEG (icEEG) obtained during clinical epilepsy monitoring. By sampling neuronal activity from cortical and deep brain structures with millisecond precision (Blount et al. 2008; Rosenow and Lüders 2001), icEEG can be acquired simultaneously with fMRI (Wilson et al. 2024; Tehrani et al. 2021; Chaudhary et al. 2021), offering a unique opportunity to examine the electrophysiological correlates of BOLD‐derived FC between sampled electrode locations.

Prior work pooling icEEG–fMRI data from nine patients reported associations between BOLD FC and electrophysiological connectivity and identified recurring connectivity patterns in both fMRI and icEEG signals that were not necessarily temporally synchronized (Mostame et al. 2025; Ridley et al. 2017). In parallel, methodological work has shown that apparent dynamic FC could result from sampling variability around time‐averaged connectivity rather than neural reconfiguration alone (Laumann et al. 2024). These findings highlight the importance of distinguishing average electrophysiological coupling across sampled connections from window‐level electrophysiological fluctuations when interpreting dynamic fMRI connectivity.

To address these questions, we analyzed simultaneously acquired resting‐state icEEG and fMRI data from 48 individuals undergoing intracranial video‐EEG monitoring. We quantified both static and dynamic icEEG–fMRI coupling across canonical frequency bands. Static analyses tested whether time‐averaged icEEG connectivity was associated with corresponding time‐averaged fMRI FC across sampled electrode‐pair connections, using both full‐recording static FC and window‐averaged FC summaries. Dynamic analyses decomposed sliding‐window icEEG connectivity into average and window‐specific deviation terms to test whether window‐level icEEG FC fluctuations were associated with temporally aligned fMRI FC after accounting for average electrophysiological coupling. Associations were quantified using hierarchical linear mixed‐effects models that accounted for repeated measures across windows, runs, electrode‐pair connections, and subjects. We further examined exploratory spatial variation in cross‐modal coupling and evaluated robustness to head motion and other methodological choices. Based on prior multimodal studies (Koch et al. 2009; Mulert et al. 2010; Scheeringa et al. 2011; Pan et al. 2011), we hypothesized that fMRI FC would show frequency‐dependent associations with electrophysiological connectivity across both static and dynamic analyses.

## 2 Materials and Methods

### 2.1 Participants

Seventy individuals undergoing intracranial video‐EEG monitoring (VEM) for drug‐resistant focal epilepsy were prospectively recruited between 2008 and 2021 for simultaneous icEEG‐fMRI acquisition. Inclusion criteria were age > 18 years, ability to provide informed consent, absence of MRI contraindications, and a clinical diagnosis of focal epilepsy. The study was approved by the University of Calgary Conjoint Health Research Ethics Board.

### 2.2 Intracranial EEG Data Acquisition

Each subject's intracranial VEM data were reviewed by an experienced epileptologist (P.F.) to select up to eight electrodes to be used during the icEEG‐fMRI acquisition. Selected electrodes were connected through an MRI‐compatible electrode connector system supporting up to 64 channels (Compumedics Neuroscan) and routed to a SynAmps RT headbox and amplifier system (Compumedics Neuroscan). EEG signals were acquired at 10 or 20 kHz using Scan 4.4 software before 2020 or Curry 8 software from 2020 onward. The EEG system clock was synchronized with the MRI scanner clock. Online artifact rejection and filtering were used to facilitate EEG monitoring during acquisition.

### 2.3 MRI Data Acquisition

Subjects were scanned using a 3 T GE Discovery MR750 whole body scanner with an eight‐channel receive‐only phased‐array head coil (GE Healthcare). Subjects were closely monitored during the acquisition. The MRI protocol included multislice anatomical imaging [spoiled gradient‐recalled echo two‐dimensional (2D) multislice sequence: echo time = 2.1 ms, repetition time = 150 ms, flip angle = 18°, 128 × 128 matrix, 24 slices 5.00 mm thick], anatomical 3D T1‐weighted imaging (echo time = 3.8 ms, repetition time = 9.3 ms, flip angle = 12°, 24‐cm field of view, 320 × 256 × 64 matrix, 0.47 × 0.47 × 2.00 mm slices), and fMRI (gradient‐recalled echo planar imaging: echo time = 30 ms, repetition time = 1500 ms, flip angle = 65°, 24‐cm field of view, 64 × 64 matrix, 24, 25, or 29 slices 3.75 × 3.75 × 5.00 mm). During resting‐state fMRI acquisition, participants were instructed to keep their eyes closed. Subjects were scanned for up to 60 min, comprising three 20‐min resting‐state fMRI runs.

### 2.4 Intracranial EEG Preprocessing

icEEG data were preprocessed using an in‐house MATLAB pipeline (R2022b; MathWorks, Natick, MA). For the purpose of gradient artifact correction, channels were re‐referenced using a within‐electrode common average to minimize gradient artifact amplitude while preserving the flexibility of a referential montage for subsequent identification of epileptiform activity. The preprocessing pipeline included: (i) bandpass filtering (0.5–500 Hz); (ii) correction of MRI gradient artifacts using average artifact subtraction (Allen et al. 2000); (iii) reduction of residual artifacts using principal component analysis across TR epochs (Niazy et al. 2005); and (iv) removal of line noise (60 Hz and harmonics) using notch filtering. Data were subsequently downsampled to 2500 Hz.

### 2.5 fMRI Preprocessing

All MRI data were processed using FEAT version 6.0, part of the FSL FMRIB Software Library (FSL; https://fsl.fmrib.ox.ac.uk/fsl/docs/). Standard preprocessing included slice‐timing correction, brain extraction (Smith 2002), head motion correction (Jenkinson et al. 2002), spatial smoothing with a 6‐mm FWHM Gaussian kernel, and high‐pass temporal filtering with sigma = 50 s. Frames with head motion exceeding 1.5 mm were removed, and affected runs were split into contiguous segments at censored frames. Segments were not concatenated across motion boundaries; rather each retained segment was treated as a separate run. Segments shorter than 5 min were excluded from further analysis.

FSL's multivariate exploratory linear optimized decomposition into independent components (MELODIC) tool was used to decompose each functional scan into 60 components. All components were visually inspected by two reviewers (T.R., W.W.) to identify noise‐ and or artifact‐related components (Griffanti et al. 2017), which were then regressed out of the data. Functional MRI data were registered to both low‐ and high‐resolution anatomical images using FMRIB linear image registration tool (FLIRT) (Jenkinson et al. 2002; Jenkinson 2001).

### 2.6 Regions of Interest (ROI) and Electrode‐to‐fMRI Mapping

Electrode contacts were manually localized on each subject's post‐implantation T1‐weighted image. Bipolar channels were defined as differences between adjacent contacts along each depth electrode and served as the basis for all analyses. Channels exhibiting interictal epileptiform discharges (IEDs) were excluded. For each remaining bipolar channel, a corresponding fMRI ROI was defined in native space by combining 3 × 3 × 3 voxel neighborhoods centered on the two constituent contacts, from which the mean BOLD time series was extracted and bandpass‐filtered between 0.01 and 0.1 Hz. Pairwise FC was then computed between bipolar icEEG channels and between their corresponding fMRI ROIs. Throughout the manuscript, a sampled electrode‐pair connection refers to a pair of bipolar icEEG channels together with the corresponding pair of fMRI ROIs. To prevent inflated correlations due to overlapping anatomical sampling, connections between bipolar channels sharing a common contact were excluded from all coupling analyses.

### 2.7 Functional Connectivity Analyses (icEEG)

Static and dynamic icEEG FC were computed to characterize time‐averaged and window‐level interactions during rest. Continuous icEEG signals were filtered into canonical frequency bands: delta: 1–3 Hz; theta: 4–7 Hz; alpha: 8–12 Hz; beta: 13–30 Hz; low gamma: 31–50 Hz; high gamma: 51–100 Hz. While higher frequencies are available in icEEG, the upper limit of 100 Hz was chosen a priori to balance sensitivity to high‐frequency icEEG activity with signal quality and interpretability in simultaneous icEEG–fMRI recordings, as higher‐frequency activity is more susceptible to lower signal‐to‐noise ratio due to residual gradient artifacts and could also be confounded by higher‐frequency oscillations (HFO)–range activity (Frauscher and Gotman 2019). This upper limit was selected a priori to balance sensitivity to high‐frequency icEEG activity with signal quality and interpretability in simultaneous icEEG–fMRI recordings. Band‐limited power (BLP) time series were obtained using the Hilbert transform in MATLAB. Full‐recording static icEEG FC was defined as the Pearson correlation between BLP time series for each pair of bipolar channels across the full retained recording. Dynamic icEEG FC was estimated using 120 s sliding windows (Leonardi and Van De Ville 2015) with 60 s overlap (18 windows per run), with windowed correlations computed for each channel pair to obtain time‐resolved FC estimates (Figure 1). A window‐averaged icEEG FC was also computed by averaging sliding‐window FC estimates across retained windows and runs, providing a whole‐scan summary based on the same windowed estimator used in the dynamic analyses.

**FIGURE 1:**
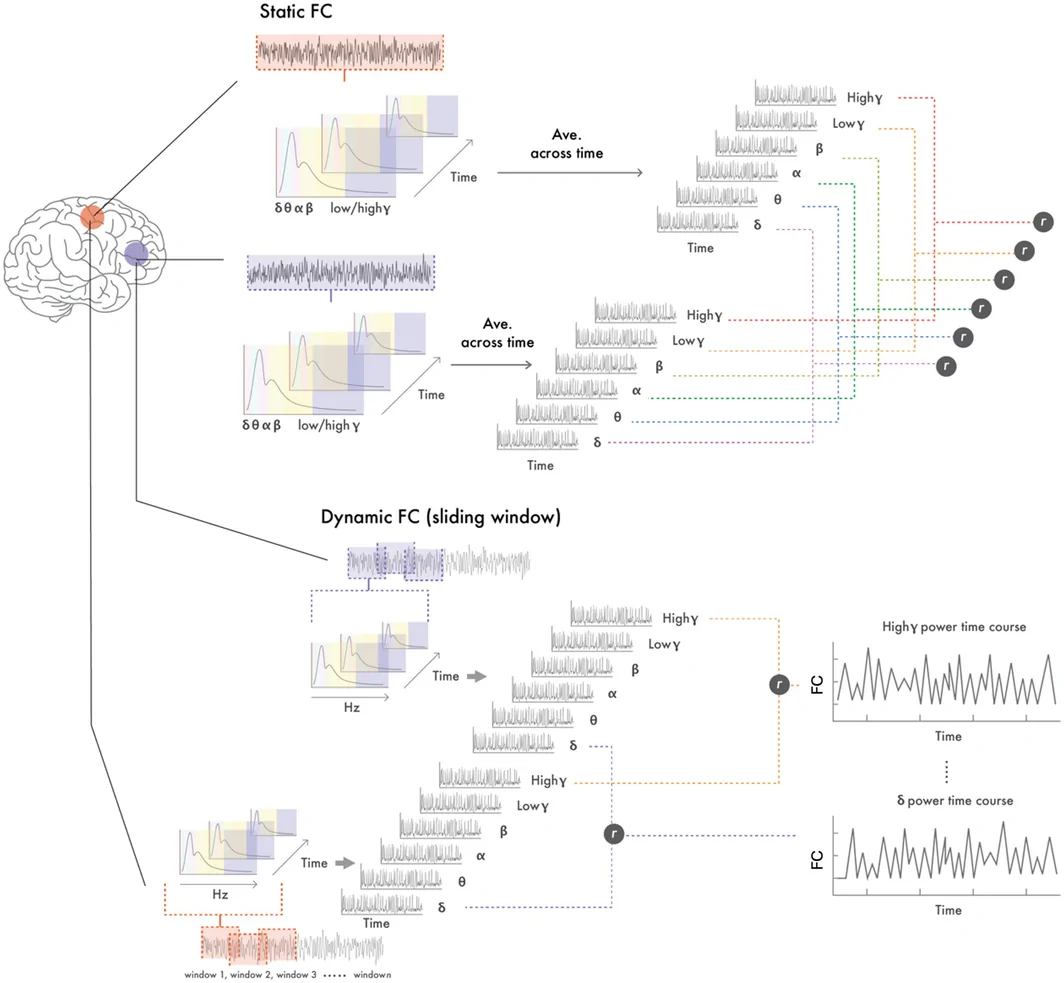
Calculation of static and dynamic functional connectivity (FC) from icEEG band‐limited power (BLP) time series. Continuous icEEG signals were filtered into canonical frequency bands, and BLP timeseries were extracted using the Hilbert transform. Static FC was computed as the Pearson correlation between BLP time series from each pair of bipolar channels across the full recording. Dynamic FC was estimated from the same BLP timeseries using a sliding‐window approach, yielding time‐resolved connectivity estimates for each channel pair.

### 2.8 Functional Connectivity Analyses (fMRI)

Static and dynamic resting‐state fMRI FC were computed using ROIs corresponding to bipolar icEEG locations. BOLD time series were extracted from ROIs corresponding to bipolar icEEG locations and shifted by 6 s, corresponding to 4 TRs, to approximate the expected hemodynamic delay. Full‐recording static fMRI FC was defined as the Pearson correlation between ROI BOLD time series across the full retained recording. Dynamic fMRI FC was estimated using the same sliding‐window framework as the icEEG analysis, with 120 s windows and 60 s overlap (Figure 2). A window‐averaged fMRI FC summary was also computed by averaging sliding‐window fMRI FC estimates across retained windows and runs. Correlation values were Fisher *z*‐transformed before statistical analysis.

**FIGURE 2:**
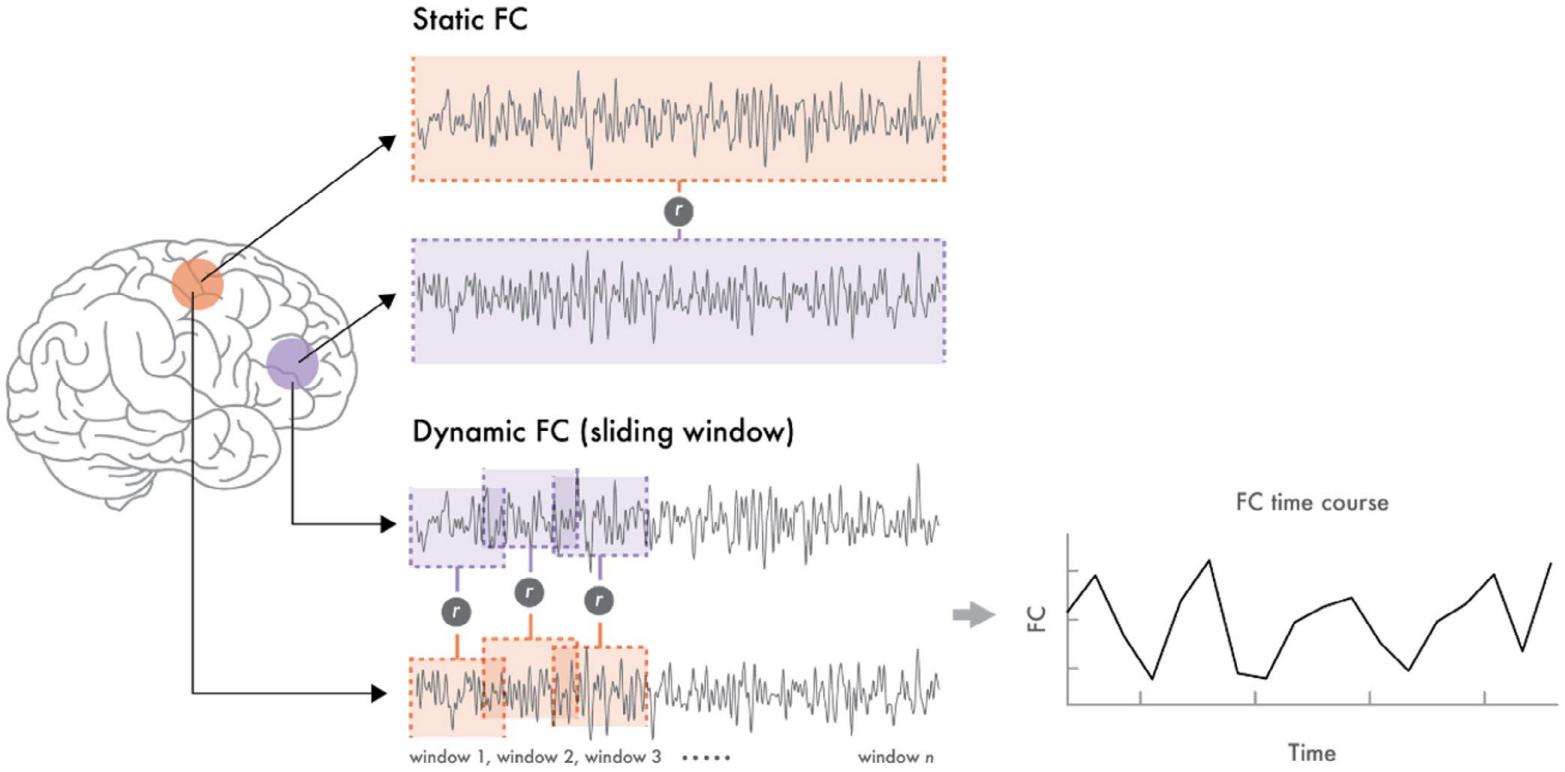
Calculation of static and dynamic functional connectivity (FC) from fMRI BOLD time series. Regional BOLD time series were extracted from regions of interest (ROIs) corresponding to bipolar icEEG locations and shifted by 6 s (four TRs) to account for the hemodynamic delay. Static FC was computed as the Pearson correlation between BOLD time series from each pair of ROIs across the full scan. Dynamic FC was estimated using a sliding‐window approach matched to the icEEG analysis, yielding time‐resolved connectivity estimates.

### 2.9 Static icEEG–fMRI Coupling Analysis

To assess stable correspondence between electrophysiological and hemodynamic connectivity, static icEEG–fMRI coupling was evaluated using the two FC summaries defined above. First, full‐recording static FC was computed over the full retained recording duration for each subject, electrode‐pair connection, and frequency band, yielding a conventional whole‐recording measure of time‐averaged connectivity. Second, window‐averaged FC was computed as the mean of sliding‐window FC estimates across retained windows and runs, yielding one mean connectivity value per modality for each electrode pair. Although derived from a windowed framework, this estimator represents a static summary of connectivity and was included to provide direct comparability with the dynamic window‐level analyses.

### 2.10 Dynamic Window‐Level icEEG–fMRI Coupling Analysis

To assess window‐level coupling between electrophysiological and hemodynamic connectivity, sliding‐window icEEG FC was decomposed into two components: (i) a subject‐, electrode‐pair‐, and frequency band‐specific mean icEEG FC term, computed as the average of sliding‐window icEEG FC estimates across retained windows and runs; and (ii) a window‐specific icEEG FC deviation term (ΔicEEG), computed as the difference between each windowed icEEG FC estimate and the corresponding mean sliding‐window icEEG FC. In other words:

ΔicEEG=windowed icEEGFC−mean windowed icEEGFC

This decomposition was used to separate average differences in icEEG FC across sampled electrode‐pair connections from fluctuations around that average within the same connection. Thus, ΔicEEG was centered by construction within each subject, electrode‐pair connection, and frequency band, and was interpreted as a measure of within‐connection window‐level variation in icEEG FC.

Specifically, window‐level icEEG FC was expressed as follows:

$${\text{icEEG}\_\mathrm{FC}}_{s,e,b,r,w}^{\mathrm{win}}={\bar{\text{icEEG}\_\mathrm{FC}}}_{s,e,b}^{\mathrm{win}}+\Delta {\text{icEEG}\_\mathrm{FC}}_{s,e,b,r,w}^{\mathrm{win}}$$

where ${\bar{\text{icEEG}\_\mathrm{FC}}}_{s,e,b}^{\mathrm{win}}$ denotes the mean sliding‐window icEEG FC for subject s, electrode‐pair connection e, and frequency band b, computed across retained windows and runs. $\Delta {\text{icEEG}\_\mathrm{FC}}_{s,e,b,r,w}^{\mathrm{win}}$ denotes the window‐specific deviation from that mean for subject s, electrode‐pair connection e, frequency band b, run r, and window w. Windowed fMRI FC for the corresponding electrode‐pair connection and time window was then modeled as a function of both the mean icEEG FC term and the window‐specific ΔicEEG term, allowing average electrophysiological coupling across sampled connections to be distinguished from window‐level electrophysiological fluctuations. Because the mean icEEG FC term and ΔicEEG were derived from the same sliding‐window icEEG FC estimates, they were not interpreted as independent measurements. Rather, this decomposition was used to partition the same windowed icEEG FC signal into an average connection‐specific component and a window‐specific deviation component. Full model equations and random‐effects specifications are provided in the Supporting Information.

### 2.11 Linear Mixed‐Effects Modeling and Statistical Inference

Associations between electrophysiological and hemodynamic connectivity were tested using linear mixed‐effects models (LMMs). Models were used to account for the hierarchical structure of the data, including repeated observations within subjects and across electrode‐pair connections, and for dynamic analyses, repeated observations across time windows.

For static icEEG–fMRI coupling, models were fitted separately for each frequency band to test whether variation in icEEG FC across sampled electrode‐pair connections was associated with corresponding variation in fMRI FC. Separate models were fitted for the full‐recording static FC estimator and the window‐averaged FC estimator. Fixed effects included the relevant icEEG FC term, icEEG FC variability where applicable, inter‐electrode distance, age, and sex. Inter‐electrode distance was log‐transformed and mean‐centered to account for systematic distance‐related effects on connectivity estimates. Random effects included subject‐ and electrode‐pair‐level terms to account for repeated observations and inter‐individual variability.

Because the dynamic analyses were performed at the window level, we first characterized the variance structure of windowed FC using intraclass correlation coefficients (ICCs). ICCs were computed separately for windowed fMRI FC and band‐limited windowed icEEG FC using random‐intercept models with subject as the grouping factor. For fMRI, duplicate rows across icEEG frequency bands were removed because the same windowed fMRI FC estimates were repeated for each band. ICCs were interpreted as the proportion of total window‐level FC variance attributable to between‐subject differences. Bootstrap standard deviations were estimated by resampling subjects with replacement 500 times.

For dynamic window‐level coupling, band‐wise models were fitted separately for each frequency band, with windowed fMRI FC for each electrode‐pair connection and temporally aligned time window as the dependent variable. Fixed effects included the mean sliding‐window icEEG FC term, the window‐specific icEEG FC deviation term (ΔicEEG), and icEEG FC variability, quantified as the standard deviation of windowed icEEG FC across retained windows and runs for each subject, electrode‐pair connection, and frequency band. Inter‐electrode distance, age, and sex were included as covariates. Random effects included electrode‐pair connection, run nested within subject, and subject‐specific random slopes for ΔicEEG. Statistical inference focused on the fixed effect of ΔicEEG, which tested whether within‐connection, window‐level deviations in icEEG FC were associated with temporally aligned windowed fMRI FC after accounting for mean icEEG FC, overall icEEG FC variability, inter‐electrode distance, age, and sex.

To formally test whether the ΔicEEG coupling effect differed across frequency bands, we fitted an additional omnibus dynamic LMM including frequency band and a band × ΔicEEG interaction term. This model was compared with a reduced model without the interaction term using a likelihood‐ratio test. Models were fitted using maximum likelihood for this comparison. This analysis tested whether the fixed effect of ΔicEEG differed across frequency bands, rather than relying only on differences in FDR‐corrected significance across separate band‐wise models.

Multiple comparisons across icEEG frequency bands were controlled using the Benjamini–Hochberg false discovery rate procedure within each analysis. Reported coefficients reflect fixed‐effect estimates from the corresponding LMMs. Full model equations, random‐effects specifications, ICC analyses, and sensitivity/control analyses are provided in the Supporting Information. An overview of the analysis is shown in Figure 3.

**FIGURE 3:**
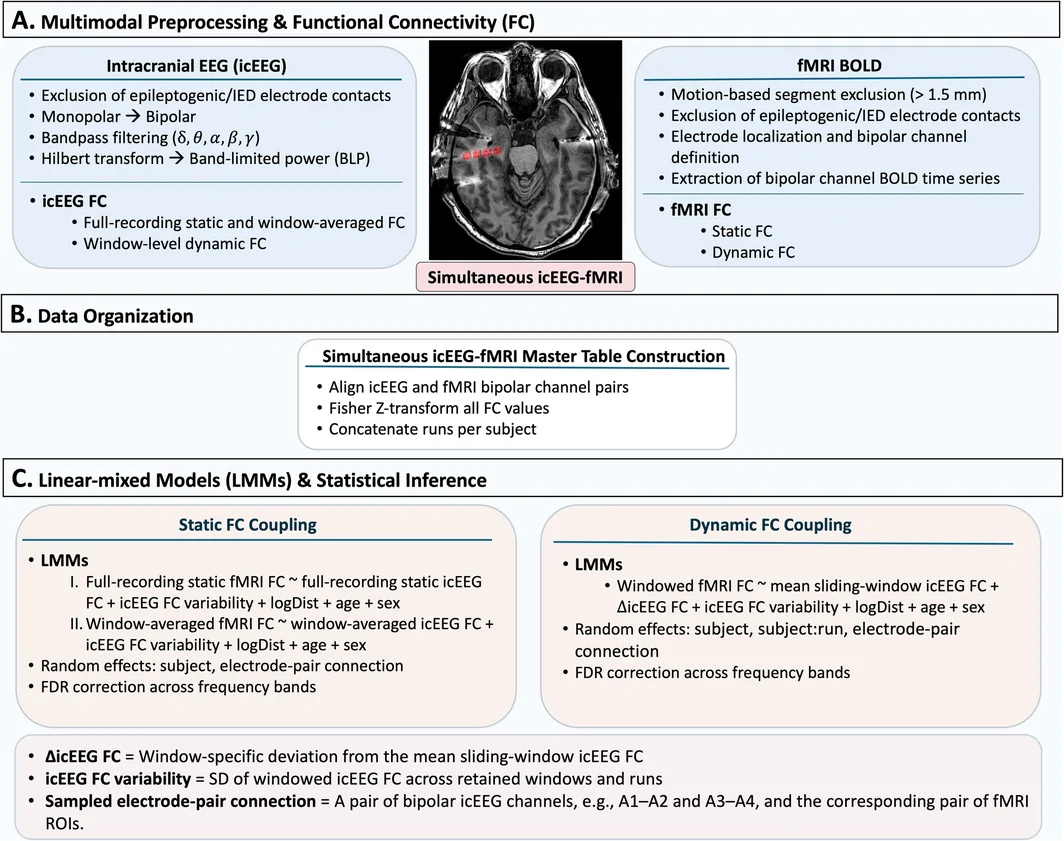
Overview of preprocessing, data integration, and statistical modeling pipeline. (A) Simultaneous intracranial EEG (icEEG) and fMRI preprocessing workflows. icEEG signals were filtered into canonical frequency bands, bipolar‐referenced, and organized into static and window‐level dynamic functional connectivity (FC) estimates. fMRI data underwent standard preprocessing including motion correction, regression of epileptogenic contacts, and static and dynamic FC computation. (B) Data organization and master table construction. icEEG and fMRI connectivity estimates were aligned at the bipolar channel‐pair level, Fisher *z*‐transformed, and concatenated within subject for integrated modeling. (C) Linear mixed‐effects modeling framework. Static models assessed associations between mean icEEG FC and mean fMRI FC, whereas dynamic models examined the contribution of window‐specific icEEG deviations (ΔEEG), static coupling, temporal variability, inter‐electrode distance, and demographic covariates to windowed fMRI connectivity, with random effects accounting for subject, run, and sampled electrode‐pair connections variability.

### 2.12 Sensitivity and Robustness Analyses

Several sensitivity and robustness analyses were performed to assess whether the reported icEEG–fMRI associations were robust to key preprocessing choices, dynamic FC parameters, anatomical sampling, and head motion. First, to assess whether the results were influenced by the 6‐mm FWHM spatial smoothing kernel used in the primary fMRI preprocessing pipeline, we repeated the icEEG–fMRI coupling analyses using fMRI data processed with alternative 4‐mm and 8‐mm FWHM spatial smoothing kernels. All other preprocessing and analysis parameters were kept identical to the primary analysis. For the static analyses, we assessed whether full‐recording static FC and window‐averaged FC associations were robust across smoothing kernels. For the dynamic analyses, statistical inference focused on the primary window‐level coupling term, ΔicEEG, while mean sliding‐window icEEG FC and icEEG FC variability were retained as covariates in the model. The 4‐mm and 8‐mm dynamic analyses were restricted to the same subject, run, sampled electrode‐pair connection, frequency band, and window keys used in the primary 6‐mm analysis. These analyses were used to assess whether the reported icEEG–fMRI coupling results were sensitive to the choice of fMRI spatial smoothing kernel.

Second, to assess sensitivity to temporal alignment between icEEG and fMRI connectivity estimates, we repeated the dynamic icEEG–fMRI coupling models using fMRI temporal shifts of 0, 3, and 9 s, in addition to the primary 6 s shift. The 6 s shift was selected a priori to approximate the expected timing of the BOLD response. Lag‐specific analyses were restricted to the same subject, run, sampled electrode‐pair connection, frequency‐band, and window keys to ensure that differences across temporal shifts reflected temporal alignment rather than changes in the analytic sample.

Third, to assess sensitivity to dynamic FC window parameters, we repeated the dynamic analyses using shorter 90 s windows with 45 s overlap, longer 150 s windows with 75 s overlap, and non‐overlapping 120 s windows. For each window setting, both icEEG FC and fMRI FC were recomputed using the corresponding window length and overlap. The primary 120 s window with 60 s overlap contained 80 fMRI volumes at TR = 1.5 s and was selected to balance temporal resolution with the reliability of correlation‐based FC estimates. Because a 0.01‐Hz BOLD fluctuation corresponds to a period of approximately 100 s, the primary 120 s window was chosen to provide sufficient temporal coverage for low‐frequency BOLD fluctuations while retaining multiple windows per run. The non‐overlapping 120 s analysis was included to assess whether the dynamic coupling results were influenced by dependence introduced by the 50% overlap between adjacent windows, because overlapping windows share data and may induce serial autocorrelation in windowed FC estimates.

Fourth, to assess whether dynamic cross‐modal coupling varied by anatomical location, the window‐level LMM framework was applied separately to sampled electrode‐pair connections grouped by anatomical region pairs. Bipolar icEEG channels were assigned to retained anatomical bins based on electrode location: LF, left frontal; LMT, left mesial temporal; LI, left insula; RF, right frontal; RMT, right mesial temporal; and RI, right insula. These regions were selected because they were sufficiently represented across the heterogeneous clinical implantation patterns. Channels outside these regions were excluded from this stratified analysis. Because this analysis involved multiple region‐ and frequency‐specific comparisons and was limited by uneven clinically determined electrode coverage, it was treated as exploratory and interpreted descriptively rather than as a confirmatory regional analysis.

Fifth, to test whether icEEG–fMRI coupling depended on anatomical correspondence between modalities, we performed a mismatched‐connection negative‐control analysis. Within each subject and frequency band, fMRI FC values were reassigned to non‐corresponding icEEG electrode‐pair connections, thereby disrupting the anatomical correspondence between electrophysiological and hemodynamic connectivity while preserving the subject‐level and frequency‐specific connectivity distributions. For the dynamic analysis, reassignment was performed within subject, run, window, and frequency band. The same LMM framework was then refit using the mismatched connection pairings. This analysis tested whether the observed coupling was specific to spatially corresponding icEEG–fMRI connections.

Finally, to assess whether the observed icEEG–fMRI coupling was influenced by head motion, we conducted motion‐control robustness analyses. As a first‐level precaution, time windows containing excessive motion, defined as framewise displacement or relative displacement greater than 1.5 mm, were excluded from all analyses. To directly address potential residual motion confounding in static fMRI FC, we repeated the full‐recording static and window‐averaged static FC models with subject‐level mean motion included as an additional covariate. Subject‐level mean motion was computed from run‐level FEAT relative displacement estimates across retained fMRI runs. In addition, to evaluate motion effects in the window‐level analyses, runs were stratified based on run‐level motion severity. For each run, the maximum relative displacement was extracted from the motion‐correction time series generated during fMRI preprocessing in FSL FEAT. Runs were classified as low motion if maximum relative displacement was less than 0.5 mm, and moderate motion if maximum relative displacement was between 0.5 mm and the exclusion threshold. The primary window‐level LMMs were then refit within motion‐defined subgroups to assess whether icEEG–fMRI coupling persisted in low‐motion data.

## 3 Results

### 3.1 Participant Inclusion and Final Analytic Sample

Of the 70 recruited participants, 22 were excluded before the final analysis. Exclusions were due to inability to satisfactorily remove gradient‐switching artifact from the icEEG recordings (*n* = 5), less than 5 min of continuous fMRI data remaining after motion correction (*n* = 3), or implantation with strip and/or grid electrodes, either alone or in combination with depth electrodes, rather than depth‐electrode coverage suitable for the present electrode‐pair analysis (*n* = 14). The final analytic sample therefore included 48 adults with focal epilepsy.

Depth electrodes (Spencer probes SD06R‐SP05X‐000/SD08R‐SP05X‐000/SD10R‐SP05X‐000; Ad‐Tech Medical Instrument Corp. Oak Creek, WI, USA) with 5‐mm inter‐contact spacing were implanted under general anesthesia based on individualized clinical assessment. Only electrodes located in non‐pathological regions, defined as regions without ictal or interictal epileptiform activity, were included in analyses. As expected for clinically determined electrode implantation, regional coverage varied across participants.

Subjects' ages ranged in age from 27 to 72 years (mean ± SD: 44.4 ± 11.3 years) and 22 of the 48 participants were females. On average, 16.6 ± 5.2 non‐epileptogenic regions of interest (ROIs) were analyzed per subject. These ROIs corresponded to electrode contacts in which no IEDs were detected during the recording. This resulted in 143 ± 84 pairwise connections per participant included in the statistical analysis. Detailed demographic and clinical information, together with the effective icEEG‐fMRI analysis duration retained for each participant after motion censoring and exclusion of segments shorter than 5 min, are reported in Table 1.

**TABLE 1:** Clinical and analysis characteristics of the 48 subjects with drug‐resistant focal epilepsy included in the final analytic sample.

ID	Age	Sex	Diagnosed SOZ	MRI lesion status	icEEG‐fMRI duration (minutes)	# of non‐epileptogenic ROIs	# of pairwise connections
13	33	M	Left temporal	None	33.75	2	1
14	49	M	Bitemporal	None	63.38	12	66
16	40	M	Left temporal	None	5	20	190
17	51	M	Right temporal	Right hippocampal sclerosis	58.75	14	91
18	59	M	Right temporal	Multiple congenital abnormalities	44.75	15	105
22	45	M	Bitemporal	Subependymal gray matter heterotopia	60	24	276
23	38	M	Right temporal	None	55	19	171
24	52	M	Left frontal	None	30.03	18	153
27	62	F	Left temporal	None	30	15	105
28	33	F	Left frontal	Cortical dysplasia	41.58	3	3
29	31	M	Right mesial temporal	None	35	15	105
30	34	F	Left frontal	None	31.55	16	120
31	33	F	Left frontal	None	12.5	22	231
33	31	M	Right temporal	None	46.63	17	136
34	39	M	Left temporal	None	33.13	17	136
35	39	F	Right frontotemporal	None	20	11	55
36	39	M	Right temporal‐insular‐orbitofrontal	Thickened cortex in frontal operculum	20	15	105
37	36	F	Right temporal	None	8.2	17	136
38	49	M	Bitemporal	Hyperintense left amygdala	10.13	29	406
39	35	F	Left mesial temporal	Cortical dysplasia	20	11	55
40	45	F	Right orbitofrontal	Hyperintense frontal lobe	58.5	12	66
41	60	F	Right temporal neocortical, insula	None	40.5	7	21
42	42	F	Right frontal/cingulate	None	60	12	66
43	54	F	Right mesial temporal, insular, orbitofrontal	Encephalomalacia frontal lobes	60	18	153
44	63	M	Bilateral	Left mesial sclerosis	50	21	210
45	72	M	Bilateral	None	60	17	136
46	45	F	Right mesial temporal, insular, orbitofrontal	Cortical thickening	20	14	91
47	29	M	Bilateral	None	46.15	24	276
48	65	F	Left mesial temporal	None	21.25	23	253
49	49	M	Left mesial temporal	Cerebral schistosomiasis	5	15	105
50	27	M	Left insular, frontal	None	43.5	11	55
51	31	M	Right insular, lateral, frontal	None	52.63	22	231
52	60	F	Right temporal, neocortical, parietal, insular	Hippocampal Sclerosis	59.38	12	66
53	62	M	Left mesial temporal	None	60	21	210
54	33	M	Right parietal, temporal	Right frontotemporal postsurgical changes	55.68	17	136
55	29	M	Bitemporal	None	55	23	253
56	32	F	Bilateral temporal, insular	Left hippocampal sclerosis	46.25	16	120
57	40	F	Right mesial temporal, insular	None	60	21	210
58	38	M	Bilateral mesial temporal	None	45	17	136
59	46	M	Bilateral hemispheric	None	32.5	17	136
60	45	F	Right mesial temporal	None	60	17	136
62	38	F	Bitemporal	Small hippocampi	32.88	16	120
63	38	F	Left frontal	None	33.13	21	210
64	44	F	Right mesial temporal, occipital	Right periventricular heterotopia	7.5	22	231
65	35	F	Left mesial temporal	None	13.75	19	171
66	69	F	Right insular	None	29.75	19	171
69	45	M	Right multifocal	Right frontal cavernoma	36.88	19	171
70	51	M	Left insular	Right hippocampal sclerosis	50	13	78

*Note:* Clinical variables include age, sex, diagnosed seizure onset zone (SOZ), and MRI lesion status based on clinical imaging. The icEEG‐fMRI duration indicates the effective resting‐state data retained for analysis after motion censoring and exclusion of segments shorter than 5 min. The number of non‐epileptogenic regions of interest (ROIs) corresponds to bipolar electrode channels included in both icEEG and fMRI analyses. The number of electrode‐pair connections represents the total number of valid connectivity pairs after excluding pairs of bipolar channels that shared a common electrode contact, thereby avoiding overlap between the corresponding fMRI ROIs.

### 3.2 Static icEEG Connectivity Is Associated With Spatial Patterns of fMRI FC Across Sampled Electrode‐Pair Connections

Linear mixed‐effects models (LMMs) revealed that mean icEEG FC was positively associated with mean fMRI connectivity across all frequency bands (Figure 4). Connections with stronger electrophysiological coupling consistently showed stronger hemodynamic coupling. When connectivity was estimated over the full retained recording duration, associations were significant in all bands (all $p_{\mathrm{FDR}}$ < 0.001; Figure 4A). Effect sizes were largest in the beta and low‐gamma ranges, with comparatively smaller but still significant effects in the delta, theta, alpha, and high‐gamma bands. Full fixed‐effect estimates and 95% confidence intervals for all static models are provided in Table S1.

**FIGURE 4:**
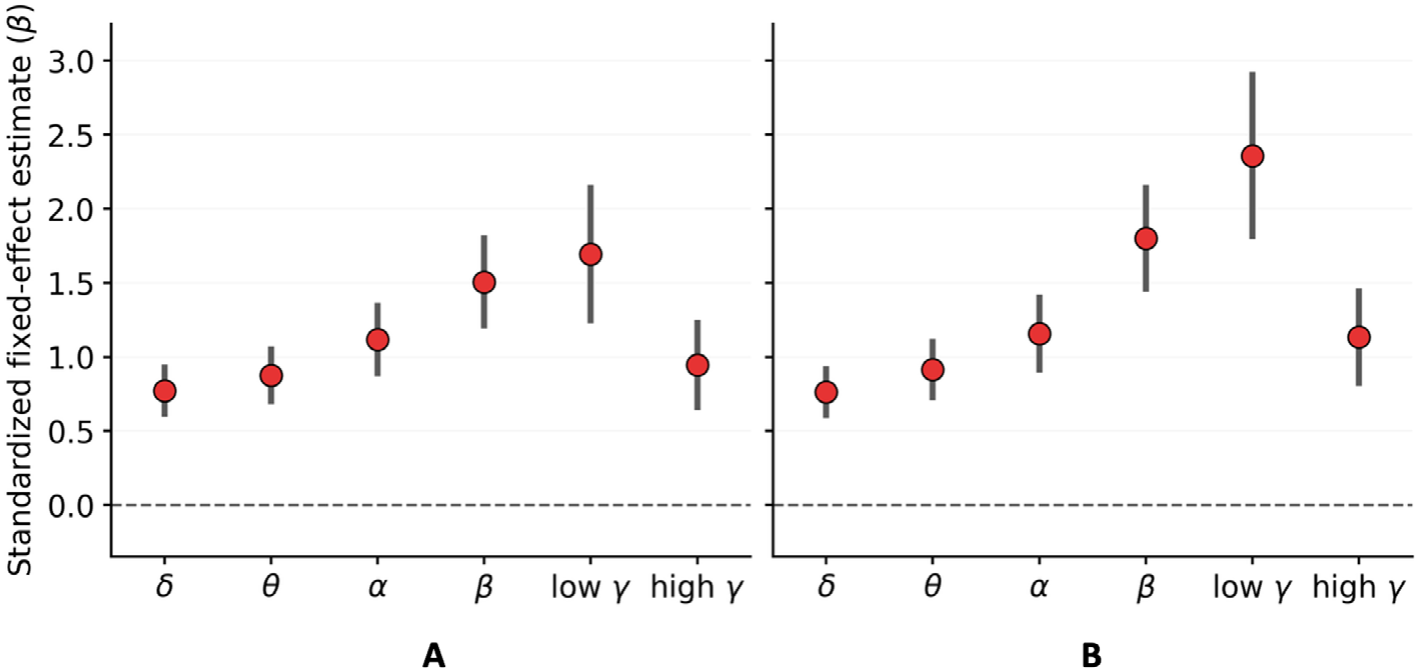
Static icEEG–fMRI coupling across frequency bands. Linear mixed‐effects model estimates quantifying the association between electrophysiological connectivity and corresponding BOLD functional connectivity (FC) across sampled electrode‐pair connections. (A) Full‐recording static icEEG connectivity, estimated over the full retained recording duration, was associated with full‐recording static fMRI FC across all frequency bands. (B) Window‐averaged icEEG connectivity was positively associated with window‐averaged fMRI FC across all frequency bands. Points denote standardized fixed‐effect regression coefficients (*β*) for the icEEG FC term, and error bars indicates 95% confidence intervals. All effects survived Benjamini‐Hochberg false discovery rate correction across bands ($p_{\mathrm{FDR}}$ < 0.001).

A complementary analysis using window‐averaged connectivity yielded a similar frequency profile, with systematically larger effect sizes (Figure 4B and Table S1; all $p_{\mathrm{FDR}}$ < 0.001). The close agreement between full‐recording static and window‐averaged estimates indicates that time‐averaged windowed connectivity provides a reliable approximation of static connectivity in this dataset, supporting its use as the mean sliding‐window component in subsequent dynamic analyses. Importantly, the positive association between mean icEEG and fMRI FC persisted after adjusting for temporal variability in icEEG connectivity (standard deviation of windowed FC), inter‐electrode distance, age, and sex.

To visualize the spatial organization underlying these statistical associations, representative static connectivity matrices from a single subject are shown in Figure 5. An anatomical rendering of the non‐epileptogenic bipolar contacts and representative pairwise connections is shown alongside the corresponding static fMRI and band‐limited icEEG FC matrices. Bipolar electrodes were ordered by hemisphere, anatomical structure, and orientation to group spatially adjacent contacts. Similar block‐structured patterns were observed across fMRI and icEEG connectivity matrices, particularly in low‐ and intermediate‐frequency bands, in line with the cohort‐level association between electrophysiological and hemodynamic connectivity. Connections between anatomically proximal regions tended to show stronger positive connectivity in both modalities; however, the association between static icEEG FC and fMRI FC remained statistically significant after accounting for inter‐electrode distance. The anatomical panel is provided to visualize the sampled electrode contacts and representative electrode‐pair connections. Statistical inference was based on the full set of eligible sampled electrode‐pair connectivity estimates.

**FIGURE 5:**
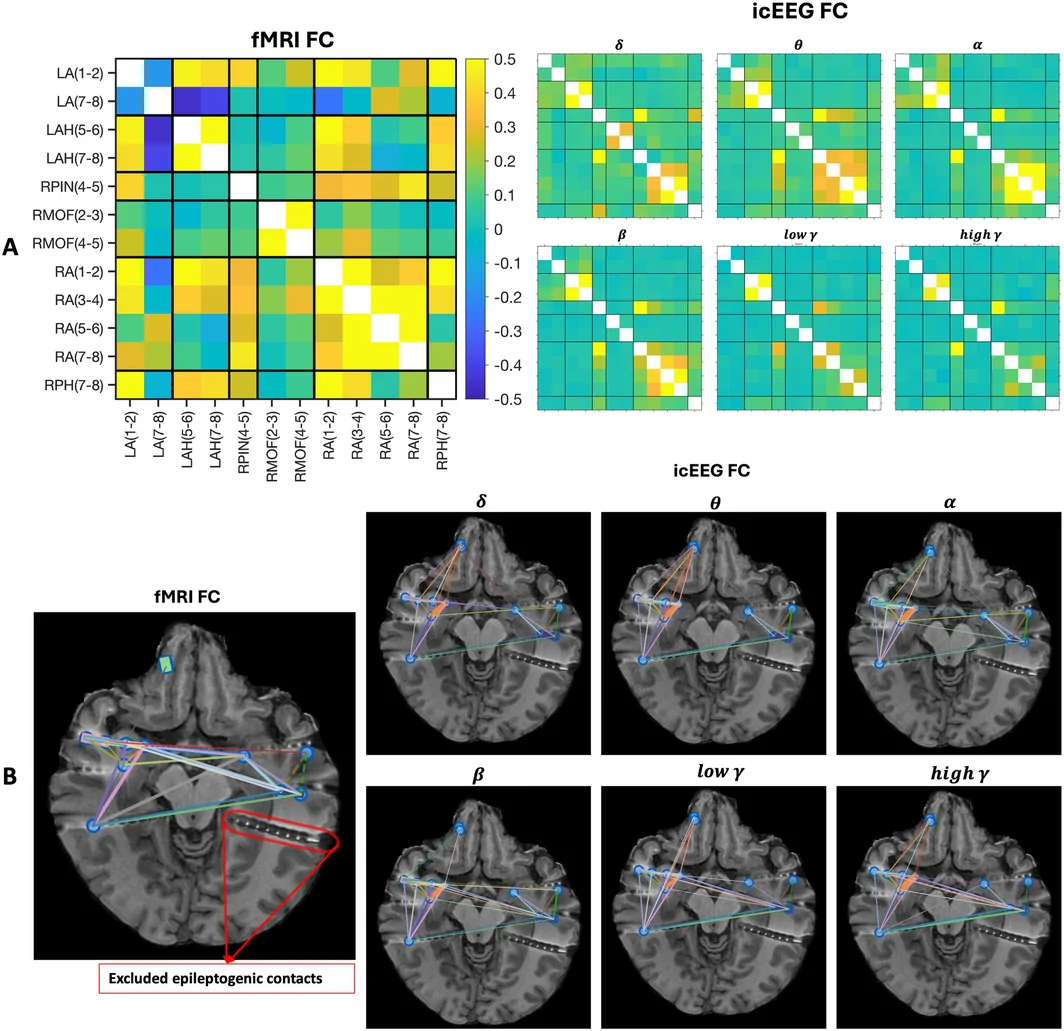
Representative static fMRI and icEEG functional connectivity (FC) across non‐epileptogenic bipolar contacts. (A) Static FC matrices from a representative subject. The large matrix on the left shows static fMRI FC, and the six matrices on the right show band‐limited static icEEG FC in the delta, theta, alpha, beta, low‐gamma, and high‐gamma bands. Each row and column corresponds to the same bipolar electrode contact across modalities and frequency bands. Electrode labels indicate hemisphere, anatomical location, and bipolar contact pair. Warmer colors indicate stronger positive FC, whereas cooler colors indicate weaker positive or negative FC. Black lines delineate anatomical electrode groupings to facilitate comparison across modalities and frequency bands. (B) Native‐space anatomical visualization of the same non‐epileptogenic bipolar contacts and representative pairwise static connections. The large anatomical panel shows fMRI static FC, and the six smaller anatomical panels show band‐limited icEEG static FC for the corresponding frequency bands. Nodes indicate bipolar contacts included in the analysis, and edges represent pairwise static FC between contacts. For visualization only, edges were thresholded to display the top 50% strongest absolute FC values, capped at 120 edges; these thresholded edges were not used for statistical inference. Solid lines indicate positive FC, and dashed lines indicate negative FC, with line thickness proportional to absolute FC magnitude. Line colors identify the same sampled electrode‐pair connection across fMRI and icEEG panels. Absence of a given color in a panel indicates that the corresponding connection was not included among the displayed thresholded edges for that modality or frequency band. Similar structured patterns across the matrices and anatomical renderings provide a representative visualization of the sampled electrode‐pair connectivity patterns quantified by the cohort‐level linear mixed‐effects models.

### 3.3 Window‐Level FC Variance Is Dominated by Within‐Subject Variability

Intraclass correlation (ICC) analysis showed that between‐subject differences accounted for a minority of the total variance in window‐level FC across both fMRI and band‐limited icEEG connectivity estimates (Table 2). For fMRI, the ICC was 0.19 ± 0.05, indicating that 19% of the total variance in windowed fMRI connectivity was attributable to between‐subject differences. Across icEEG frequency bands, ICC values ranged from 0.21 in the theta band to 0.30 in the low‐ and high‐gamma bands. Although ICC increased modestly with frequency in icEEG, values remained well below 0.5 in all bands. Thus, most variance in window‐level connectivity was attributable to within‐subject variation across sampled electrode‐pair connections, runs, windows, and residual variability rather than stable between‐subject differences.

**TABLE 2:** Intraclass correlation coefficients of window‐level functional connectivity.

Modality/band	ICC	SD
fMRI	0.19	0.05
Delta	0.24	0.07
Theta	0.21	0.04
Alpha	0.25	0.07
Beta	0.26	0.05
Low gamma	0.3	0.06
High gamma	0.3	0.07

*Note:* ICC values quantify the proportion of total variance in windowed FC attributable to between‐subject differences, based on random‐intercept models with subject as the grouping factor. The remaining variance reflects within‐subject variation across sampled electrode‐pair connections, runs, windows, and residual variability. Values are reported for fMRI and each icEEG frequency band. SD denotes the standard deviation of bootstrap ICC estimates.

These findings indicate that window‐level connectivity primarily captures within‐subject variation rather than stable trait‐like differences between participants. This variance structure supports the use of window‐level linear mixed‐effects models to examine cross‐modal coupling while accounting for repeated windows, electrode‐pair connections, runs, and subject‐level variability.

### 3.4 Dynamic fMRI Connectivity Shows Band‐Wise Associations With Window‐Level icEEG Fluctuations

The primary dynamic window‐level LMMs included 216,393 window‐level observations from 4850 sampled electrode‐pair connections across 48 subjects. An omnibus dynamic LMM including a band × window‐specific icEEG FC deviation interaction did not reach conventional significance when compared with a reduced model without the interaction term, although the result suggested possible band‐wise variation in the dynamic icEEG–fMRI coupling effect (likelihood‐ratio test: LR = 9.64, Δdf = 5, p = 0.086). In the primary band‐wise models, window‐specific icEEG FC deviations $\left(\text{ΔicEEG}\right)$ showed significant positive associations with temporally aligned fMRI connectivity in the alpha, beta, and low‐gamma bands. After FDR correction, significant positive dynamic coupling was observed in the alpha band (*β* = 0.085, 95% CI [0.026, 0.144], $p_{\mathrm{FDR}}$ = 0.015) and beta band (*β* = 0.126, 95% CI [0.038, 0.215], $p_{\mathrm{FDR}}$ = 0.015), and low‐gamma band (*β* = 0.113, 95% CI [0.023, 0.203], $p_{\mathrm{FDR}}$ = 0.028). Delta, theta, and high‐gamma bands did not survive FDR correction in the primary band‐wise models (Figure 6A). Together, these results indicate positive window‐level icEEG–fMRI coupling, with the strongest primary band‐wise evidence in alpha, beta, and low‐gamma bands, but without statistically significant omnibus evidence for differences across frequency bands.

**FIGURE 6:**
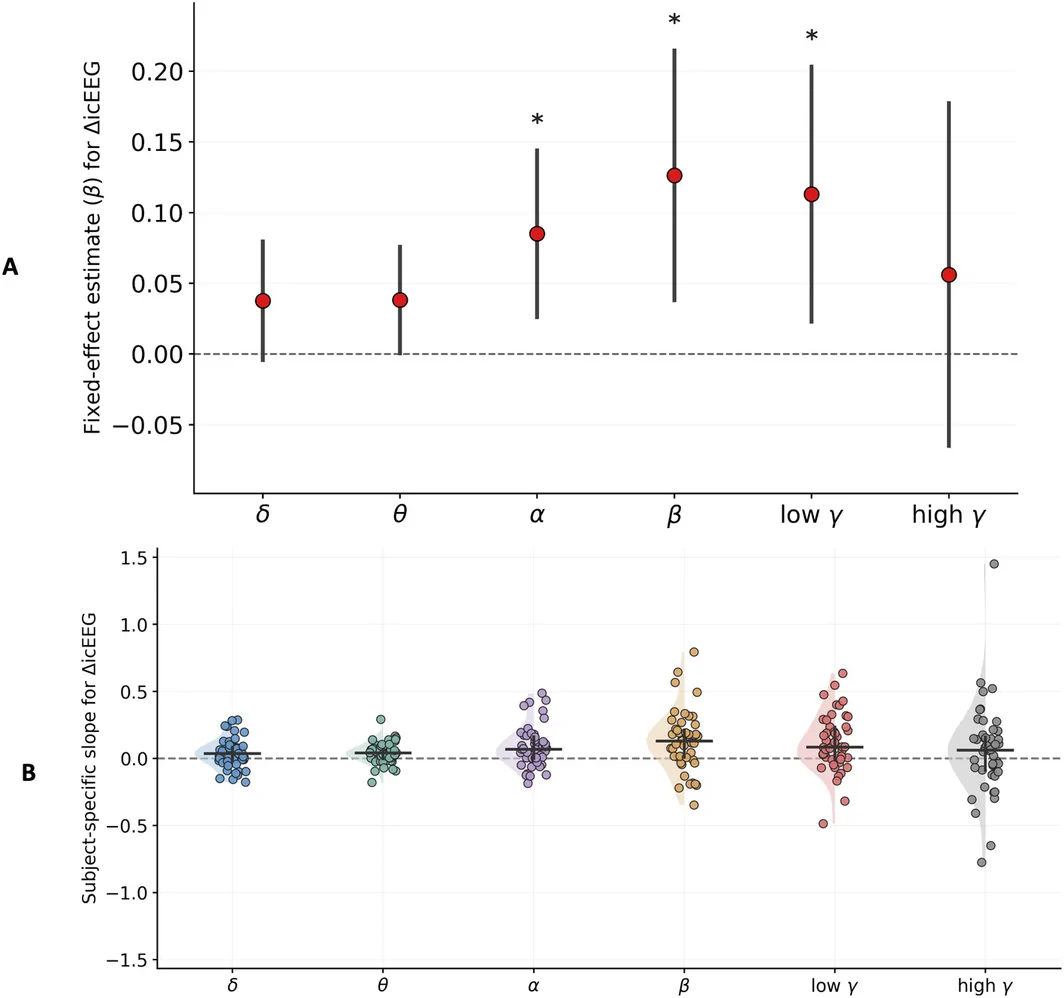
Band‐wise dynamic icEEG–fMRI coupling at group and subject levels. (A) Linear mixed‐effects model estimates quantifying the association between window‐specific icEEG FC deviations (ΔicEEG) and temporally aligned windowed fMRI connectivity across frequency bands. ΔicEEG was defined as the deviation of windowed icEEG FC from the mean sliding‐window icEEG FC for the same subject, electrode‐pair connection, and frequency band. Points denote fixed‐effect estimates (*β*), and error bars indicate 95% confidence intervals. Asterisks mark effects surviving Benjamini–Hochberg false discovery rate correction across frequency bands ($p_{\mathrm{FDR}}$ < 0.05). Significant positive associations were observed in the alpha, beta, and low‐gamma bands. The dashed horizontal line indicates zero effect. (B) Distribution of subject‐specific dynamic coupling slopes for ΔicEEG across frequency bands. Each dot represents one subject. Horizontal bars indicate the median, and vertical bars indicate the interquartile range. Positive values indicate that windows with higher‐than‐average icEEG FC tended to show stronger temporally aligned fMRI FC, whereas negative values indicate an inverse relationship.

In the same window‐level models, the mean sliding‐window icEEG FC term remained positively associated with dynamic fMRI connectivity across all frequency bands (all $p_{\mathrm{FDR}}$ < 0.001). This indicates that electrode‐pair connections with stronger average electrophysiological coupling also showed stronger hemodynamic coupling, even after accounting for window‐specific icEEG FC deviations, icEEG FC variability, inter‐electrode distance, age, and sex. The icEEG FC variability term was also associated with dynamic fMRI connectivity across frequency bands. This association was negative in the delta band and positive in theta, alpha, beta, low‐gamma, and high‐gamma bands, suggesting that the overall magnitude of electrophysiological connectivity fluctuations contributed to window‐level coupling patterns in a band‐specific sign pattern.

Allowing subject‐specific slopes for the window‐specific icEEG FC deviation term (ΔicEEG) revealed that while there was a consistent positive group‐level effect, the strength of dynamic icEEG–fMRI coupling varied across individuals. Subject‐specific slope estimates (Figure 6B) showed that although the primary band‐wise group‐level effects were significant in the alpha, beta, and low‐gamma bands, the magnitude and spectral prominence of coupling differed across participants. Some participants also showed negative slopes in specific frequency bands, indicating that, in those individuals, window‐specific icEEG FC deviations and temporally aligned fMRI FC fluctuations tended to vary in opposite directions. Representative examples (Figure 7) illustrate this variability, with one subject showing a greater magnitude of coupling in the beta band and another in the alpha band. These findings indicate that while window‐level cross‐modal dynamic coupling was evident at the group level, its expression varied across individuals and frequency bands.

**FIGURE 7:**
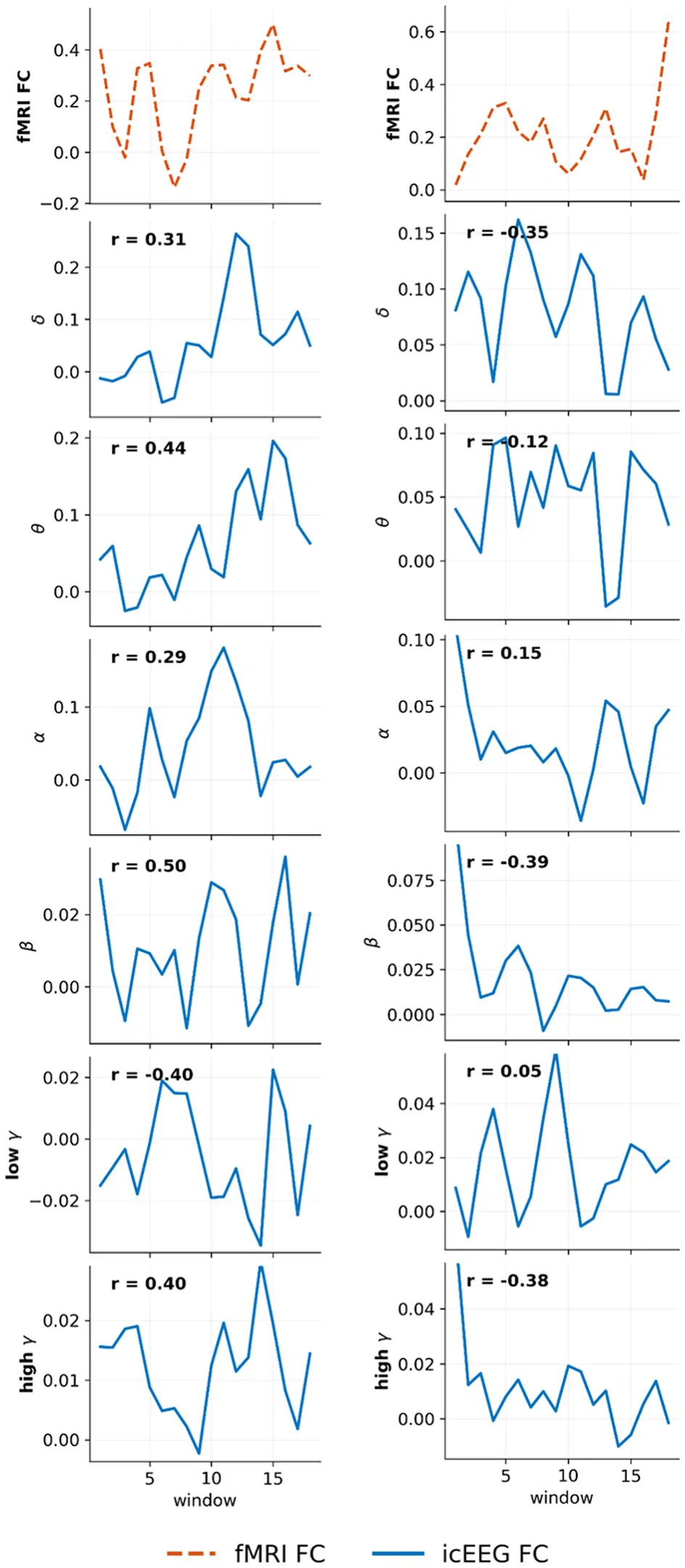
Representative window‐level correspondence between fMRI and band‐limited icEEG functional connectivity (FC). Each column shows one representative subject and sampled electrode‐pair connection. The top panel in each column shows the sliding‐window fMRI FC time course across 18 windows. The lower panels show the corresponding band‐limited icEEG FC time courses for the same electrode‐pair connection and temporally aligned windows in the delta, theta, alpha, beta, low‐gamma, and high‐gamma bands. Pearson correlation coefficients between windowed fMRI FC and band‐limited windowed icEEG FC are shown in each icEEG panel to illustrate representative subject‐, connection‐, and band‐specific correspondence. Traces are plotted using separate y‐axis scales to emphasize within‐band temporal fluctuations and should not be interpreted as between‐band amplitude comparisons. These examples are provided for visualization only; group‐level inference was based on the window‐level linear mixed‐effects models shown in Figure 6.

### 3.5 Exploratory Spatially Stratified Dynamic icEEG–fMRI Coupling

Exploratory spatially stratified dynamic icEEG–fMRI coupling. To assess spatial variation in cross‐modal coupling, the window‐level LMM analysis was repeated separately for sampled electrode‐pair connections grouped by anatomical region pairs. The resulting 6 × 6 matrices summarize the estimated coupling coefficient (*β* for ΔicEEG) for each retained region combination, where diagonal entries represent within‐region interactions and off‐diagonal entries represent between‐region interactions. Because this analysis involved multiple region‐ and frequency‐specific comparisons with uneven, clinically determined electrode coverage, regional effects are reported descriptively and were not used to support confirmatory anatomical claims.

Of the 36 possible region–region combinations displayed in the final retained anatomical bins, 28 met predefined inclusion criteria (≥ 6 subjects, ≥ 5 unique electrode pairs, ≥ 500 window‐level observations) and were retained for analysis, whereas 8 combinations (22%) were excluded a priori because of insufficient data coverage. The left and right lateral temporal bins were not retained in the final visualization because they did not meet analysis criteria in any frequency band. This partial coverage reflects clinically driven implantation patterns, with mesial temporal regions more densely sampled than several other regions. The number of contributing subjects, sampled electrode‐pair connections, and window‐level observations for each region pair are reported in Table S6.

The exploratory spatially stratified analysis revealed heterogeneous dynamic icEEG–fMRI coupling patterns across anatomical region pairs and frequency bands (Figure 8). All analyzed cells are colored according to the magnitude and direction of *β*, with asterisks indicating nominal uncorrected significance levels and n.s. indicating analyzed region pairs that did not pass the nominal threshold. Across all bands and analyzed region pairs, 11 region‐frequency cells showed nominal effects at *p* < 0.05, whereas 157 analyzed cells did not pass the nominal threshold. Positive estimates indicate that windows with higher‐than‐average icEEG connectivity for a given sampled electrode‐pair connection tended to show stronger temporally aligned fMRI connectivity, whereas negative estimates indicate the opposite pattern.

**FIGURE 8:**
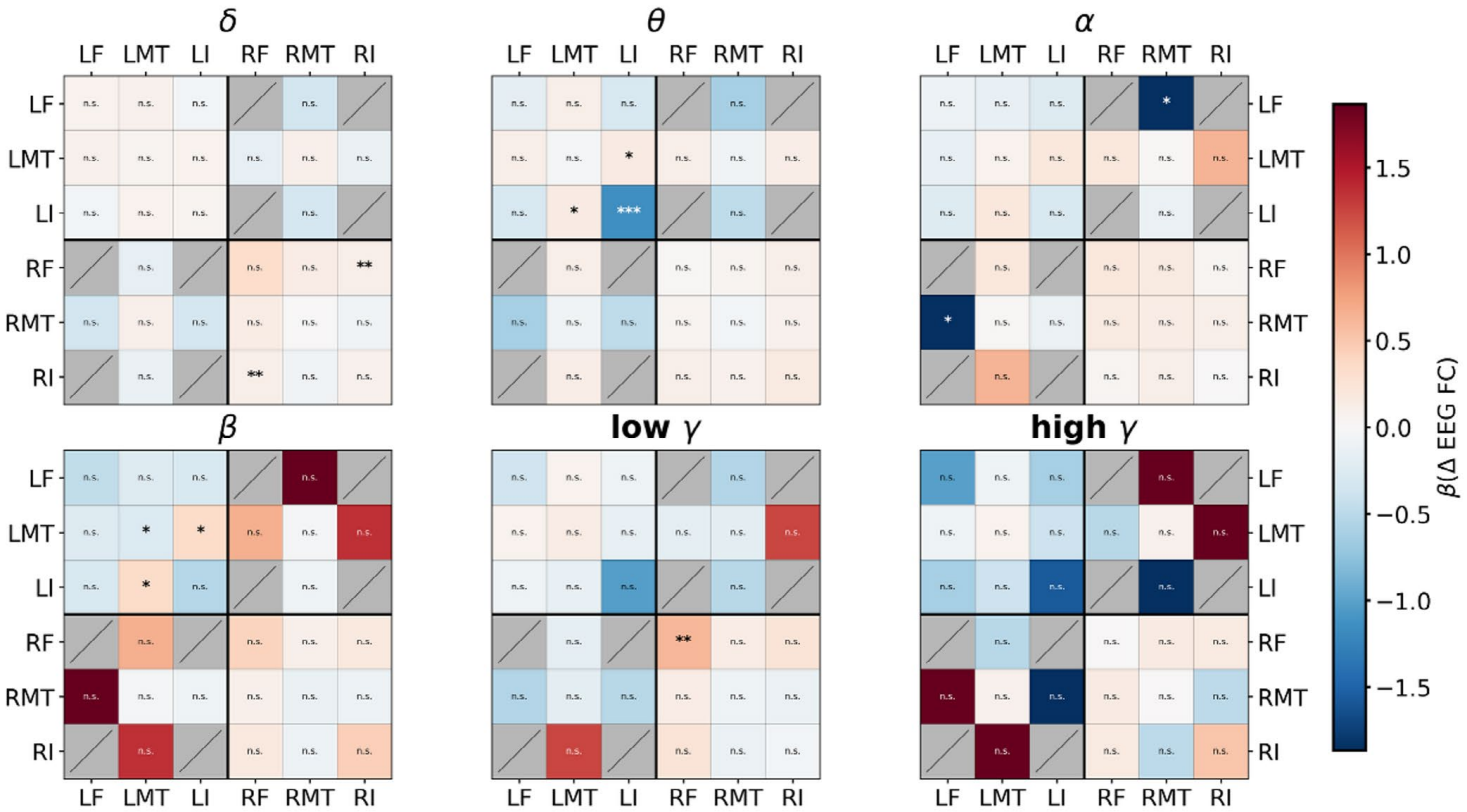
Exploratory spatially stratified dynamic icEEG–fMRI coupling across frequency bands. Each panel displays region‐by‐region coupling coefficients (*β*) for the window‐specific icEEG FC deviation term (ΔicEEG), estimated using window‐level linear mixed‐effects models. Frequency bands are arranged as delta, theta, and alpha in the top row, and beta, low‐gamma, and high‐gamma in the bottom row. Rows and columns correspond to retained anatomical bins ordered by hemisphere: LF, left frontal; LMT, left mesial temporal; LI, left insula; RF, right frontal; RMT, right mesial temporal; and RI, right insula. All analyzed cells are colored according to the magnitude and direction of *β*. Asterisks indicate nominal uncorrected significance levels (* < 0.05, **p < 0.01, ***p < 0.001), whereas n.s. indicates analyzed region pairs that did not pass the nominal threshold. Dark gray cells with diagonal slashes indicate region pairs that did not meet predefined inclusion criteria and were therefore not analyzed. Positive estimates indicate that windows with higher‐than‐average icEEG connectivity for a given sampled electrode‐pair connection tended to show stronger temporally aligned fMRI connectivity, whereas negative estimates indicate the opposite pattern. Values reflect the strength of dynamic cross‐modal coupling rather than FC magnitude. This exploratory analysis was used for descriptive visualization only and was not used to support confirmatory region‐specific claims.

Across bands, nominal effects were observed in several frontal, mesial temporal, and insular region combinations, with both positive and negative estimates. These patterns should be interpreted cautiously because the analysis was exploratory, unevenly sampled across anatomical regions, and not corrected for all region‐ and frequency‐specific comparisons. Overall, this exploratory analysis suggests that dynamic icEEG–fMRI coupling may vary across implanted anatomical regions and frequency bands, but it should not be interpreted as confirmatory evidence for specific region‐pair effects.

### 3.6 Sensitivity and Robustness Analyses

Static and window‐averaged icEEG–fMRI coupling remained positive and FDR‐significant across frequency bands when the analysis was repeated using alternative 4‐mm and 8‐mm FWHM smoothing kernels, indicating that average cross‐modal coupling was robust to the choice of spatial smoothing kernel (Table S2A). For the window‐level dynamic models, the 4‐ and 8‐mm analyses were restricted to the same subject, run, electrode‐pair connection, frequency‐band, and window keys used in the primary 6‐mm analysis. The window‐specific icEEG FC deviation term (ΔicEEG) remained positive across all frequency bands in both sensitivity analyses, but the alpha, beta, and low‐gamma effects that survived FDR correction in the primary 6‐mm analysis did not survive correction in the 4‐mm or 8‐mm analyses. Thus, the primary dynamic ΔicEEG effects were more smoothing‐sensitive than the static and average coupling effects (Table S2B).

Temporal‐alignment sensitivity analysis showed that the primary dynamic icEEG–fMRI coupling effects were consistent in direction across fMRI temporal shifts of 0, 3, 6, and 9 s (Table S3). After restricting all lag‐specific analyses to the same subject, run, sampled electrode‐pair connection, frequency‐band, and window keys, ΔicEEG effects were positive across all frequency bands and all temporal shifts. The strongest and most consistent statistical evidence was observed in the alpha, beta, and low‐gamma bands, which remained FDR‐significant at the 3, 6, and 9 s shifts, whereas the corresponding 0 s effects were positive but did not survive FDR correction. Delta, theta, and high‐gamma effects were also positive across temporal shifts but did not survive FDR correction. These results indicate that the direction of the dynamic icEEG–fMRI coupling effect was not dependent on a single assumed temporal alignment, although statistical significance was strongest when fMRI windows were shifted to account for the expected BOLD delay.

Window‐parameter sensitivity analysis showed that the direction of the primary dynamic icEEG–fMRI coupling effects was consistent across alternative window definitions (Table S4). ΔicEEG effects were positive across all frequency bands when dynamic FC was recomputed using 90 s windows with 45 s overlap, the primary 120 s windows with 60 s overlap, 150 s windows with 75 s overlap, and non‐overlapping 120 s windows. The strongest and most consistent statistical evidence was observed in the alpha, beta, and low‐gamma bands, which survived FDR correction in the 90 s, primary 120 s, and 150 s overlapping‐window analyses. In the non‐overlapping 120 s analysis, effects remained positive across all bands but did not survive FDR correction. Theta also reached FDR significance only in the 150 s window analysis, whereas delta and high‐gamma effects did not survive FDR correction under any window setting. Overall, these results indicate that the direction of dynamic icEEG–fMRI coupling was not dependent on a single window length or overlap choice, although statistical significance was strongest for overlapping‐window analyses.

In the mismatched electrode‐pair negative‐control analysis, disrupting the anatomical correspondence between icEEG and fMRI sampled electrode‐pair connections eliminated the static coupling effects and strongly attenuated the dynamic effects (Table S5). Static mismatched‐connection associations were near zero and did not survive FDR correction in any frequency band. Dynamic mismatched‐connection effects were also substantially smaller than the primary anatomically matched effects and did not survive FDR correction in any frequency band. Together, these findings indicate that the observed static and dynamic icEEG–fMRI coupling depended on anatomical correspondence between sampled icEEG and fMRI electrode‐pair connections.

To evaluate whether dynamic icEEG–fMRI coupling was influenced by head motion, we repeated the primary window‐level analyses separately within low‐motion and moderate‐motion runs (Figure 9). Across frequency bands, coupling coefficients remained positive and comparable in magnitude within the low‐motion subgroup. Confidence intervals substantially overlapped between motion strata. Importantly, coupling estimates were numerically stronger under low‐motion conditions, with no evidence of attenuation relative to higher‐motion segments. These findings indicate that the observed cross‐modal coupling effects are unlikely to be driven by residual head motion artifacts and may in fact be more robust when motion is minimized.

**FIGURE 9:**
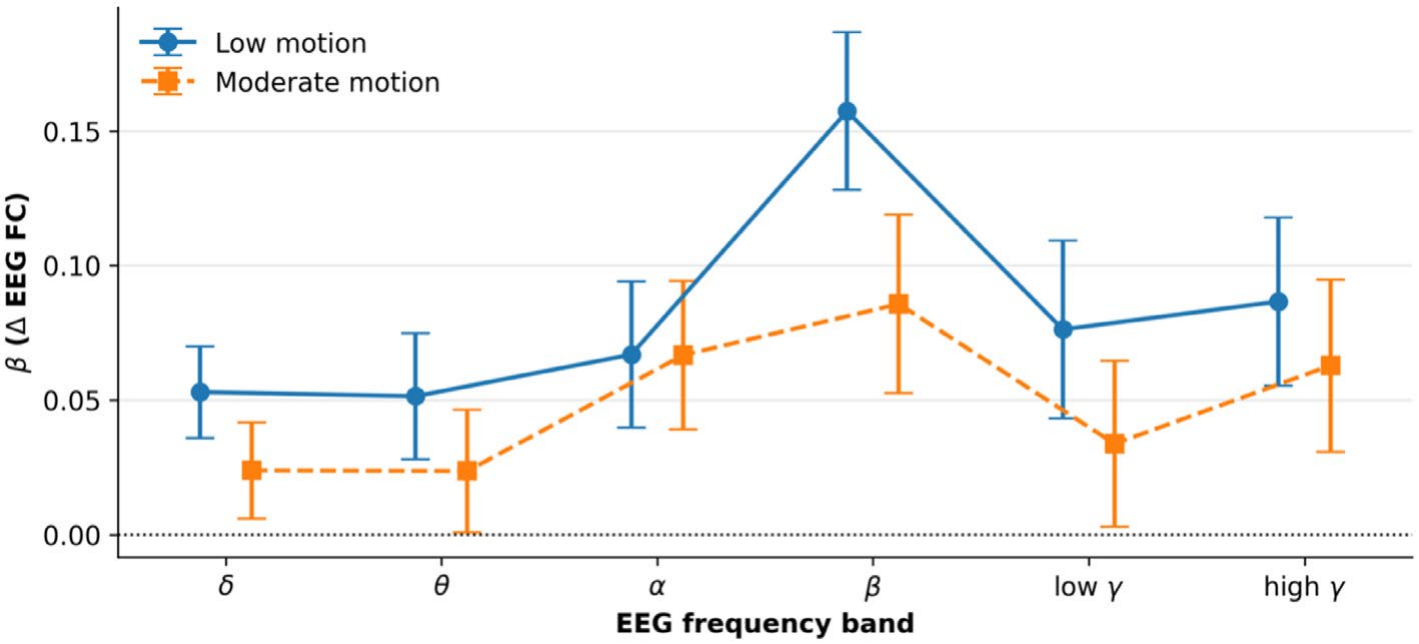
Motion robustness of dynamic icEEG–fMRI coupling. Dynamic EEG–fMRI coupling coefficients (*β* for ΔicEEG FC) estimated from window‐level linear mixed‐effects models are shown separately for low‐motion and moderate‐motion runs across EEG frequency bands. Points indicate fixed‐effect estimates and error bars represent 95% confidence intervals. Coupling effects remain positive and comparable in magnitude across motion strata, with overlapping confidence intervals, indicating that the observed cross‐modal coupling is not driven by residual head motion.

In an additional motion‐adjusted sensitivity analysis, the static icEEG–fMRI coupling effects remained positive and FDR‐significant across all frequency bands after including subject‐level mean motion as an additional covariate for both full‐recording static FC and window‐averaged static FC models (Table S7). These results indicate that the static coupling findings were not explained by residual head motion.

## 4 Discussion

Using simultaneous icEEG–fMRI, we tested how electrophysiological connectivity relates to both static and dynamic BOLD FC across frequency bands. The main finding was that fMRI FC was strongly associated with average electrophysiological coupling across sampled electrode‐pair connections and also showed positive window‐level associations with within‐connection icEEG FC deviations. Static icEEG connectivity was robustly associated with corresponding BOLD FC across sampled electrode‐pair connections, whereas dynamic fMRI connectivity showed the strongest primary band‐wise evidence of association with window‐specific icEEG FC deviations in the alpha, beta, and low‐gamma bands, with sensitivity analyses supporting directional consistency but more variable statistical significance.

These findings are consistent with prior electrophysiological evidence suggesting that slow BOLD fluctuations reflect temporally integrated neuronal activity rather than isolated transient electrophysiological events (Deco et al. 2012; Sadaghiani et al. 2010; Raichle 2010; Korhonen et al. 2014; Vanhatalo et al. 2004). Although resting‐state fMRI connectivity is susceptible to non‐neuronal influences, including physiological fluctuations and head motion (Hutchison, Womelsdorf, Gati, et al. 2013; Tong et al. 2015; Laumann et al. 2017; Lehmann et al. 2017; Chang et al. 2013; Power et al. 2014; Murphy et al. 2009), the present results support the view that electrophysiological coupling contributes to both static BOLD connectivity and, to a lesser extent, time‐resolved BOLD connectivity.

### 4.1 Static Electrophysiological Coupling Is Associated With Spatial Variation in BOLD Connectivity

Across all frequency bands, time‐averaged icEEG connectivity was significantly associated with static fMRI connectivity. Connections with stronger electrophysiological coupling exhibited stronger haemodynamic coupling, even after controlling for inter‐electrode distance, age, sex, and residual head motion in sensitivity analyses. This broadband correspondence indicates that spatial variation in resting‐state BOLD connectivity is closely related to spatial variation in electrophysiological coupling across sampled electrode‐pair connections. Importantly, the association was preserved whether connectivity was computed across the full retained recording or obtained by averaging windowed estimates, indicating that the spatial correspondence was not simply a consequence of the windowing procedure. Rather, haemodynamic connectivity appears to reflect electrophysiological coupling accumulated over time across ongoing activity (Deligianni et al. 2014; Magri et al. 2012; He et al. 2008; De Pasquale et al. 2010; Pfurtscheller and Lopes Da Silva 1999).

### 4.2 Window‐Level Electrophysiological Fluctuations Show Band‐Wise Associations With Dynamic BOLD Connectivity

Beyond static coupling, window‐specific deviations in icEEG connectivity from the mean sliding‐window icEEG FC were associated with temporally aligned fMRI connectivity in the primary band‐wise models. In these models, significant positive dynamic coupling was observed in the alpha, beta, and low‐gamma bands after FDR correction, whereas delta, theta, and high‐gamma bands did not survive correction. However, an omnibus band × ΔicEEG interaction did not reach conventional significance, although it suggested possible band‐wise variation in the dynamic coupling effect. Therefore, the band‐wise findings should not be interpreted as statistically confirmed evidence for differences between frequency bands. Rather, the results indicate that dynamic BOLD FC showed the strongest primary band‐wise evidence of association with window‐specific icEEG FC deviations in the alpha, beta, and low‐gamma bands. These effects were estimated after accounting for mean sliding‐window icEEG FC, overall icEEG FC variability, inter‐electrode distance, age, sex, and subject‐specific variability in the ΔicEEG effect.

Interestingly, the presence of negative subject‐specific slopes in a few subjects indicates that dynamic icEEG–fMRI coupling was not uniformly positive across individuals. In these participants and frequency bands, window‐specific icEEG FC deviations and temporally aligned fMRI FC tended to vary in opposite directions. These inverse associations may reflect heterogeneity in anatomical sampling, inter‐individual variability in neurovascular coupling, differences in vigilance or brain state, medication and disease‐related effects, or subject‐specific signal‐to‐noise differences. Because the present study was not designed to classify participants based on slope direction, these negative slopes should be interpreted cautiously. Future work with larger samples and more systematic anatomical sampling will be needed to determine whether inverse dynamic coupling reflects meaningful physiological subtypes or measurement variability.

Prior invasive electrophysiological studies in non‐human primates established that local field potential activity is closely related to BOLD responses, providing foundational evidence for the neuronal basis of the BOLD signal (Logothetis et al. 2001). However, those studies primarily addressed local electrophysiological activity in relation to regional BOLD amplitude, whereas the present study examined inter‐regional connectivity in simultaneous icEEG and fMRI. In addition, our high‐gamma analysis was restricted to 51–100 Hz, which should be interpreted as a restricted high‐gamma range rather than broadband high‐gamma activity extending to 180–200 Hz or higher. We therefore interpret the absence of robust dynamic coupling in the 51–100 Hz window‐specific icEEG FC deviation term cautiously. This finding does not rule out a relationship between broadband high‐gamma activity and BOLD responses but indicates that, within the frequency range and sampled electrode‐pair connectivity framework analyzed here, dynamic icEEG–fMRI coupling showed the strongest primary band‐wise evidence for dynamic icEEG–fMRI coupling observed in the alpha, beta, and low‐gamma bands.

The alpha‐band result should be distinguished from prior reports of alpha–BOLD anticorrelation (Goldman et al. 2002; Laufs et al. 2003). These simultaneous scalp EEG–fMRI studies were conducted during eyes‐closed rest and primarily examined regional alpha power in relation to local BOLD amplitude. Although both studies reported negative associations between alpha power and cortical BOLD signal, positive alpha‐related BOLD changes have also been reported in thalamic and insular regions (Goldman et al. 2002). In contrast, the present study examined inter‐regional icEEG connectivity in relation to inter‐regional fMRI connectivity across sampled electrode‐pair connections. Therefore, the positive alpha‐band icEEG–fMRI connectivity association observed here does not contradict prior alpha–BOLD anticorrelation findings, but instead suggests that alpha–BOLD relationships may depend on the electrophysiological feature measured, the spatial scale of analysis, and whether local amplitude or inter‐regional connectivity is examined. Because cognitive state, vigilance, arousal, and behavioral task demands were not directly measured, we do not infer specific functional roles for the alpha‐band coupling observed here.

Delta‐ and theta‐band window‐specific icEEG FC deviations did not show robust dynamic coupling with temporally aligned fMRI FC in the primary band‐wise models. We do not interpret this as evidence that low‐frequency activity is unrelated to BOLD dynamics. Rather, sensitivity to low‐frequency coupling may depend on features of the present windowed FC framework, including the use of correlation‐based connectivity estimates over finite time windows, the focus on electrode‐pair band‐limited power‐correlation FC rather than local power–BOLD amplitude coupling, and the use of fixed temporal alignments between icEEG and fMRI. Low‐frequency effects may also occur over slower timescales than those captured by the present window‐level deviation analysis. Thus, the non‐significant delta/theta findings should be interpreted as specific to the present windowed FC framework rather than as evidence against low‐frequency neural contributions to BOLD activity.

The mismatched electrode‐pair negative‐control analysis further supports the anatomical specificity of the observed cross‐modal associations. When the correspondence between sampled icEEG and fMRI electrode‐pair connections was disrupted within subjects, static coupling effects were eliminated and dynamic ΔicEEG effects were strongly attenuated. This indicates that the observed icEEG–fMRI coupling was not simply driven by subject‐level differences or overall connectivity distributions, but instead depended on anatomically corresponding connectivity estimates across modalities.

### 4.3 Exploratory Spatial Variation of Dynamic Coupling Across Implanted Regions

The spatially stratified analysis suggested that dynamic icEEG–fMRI coupling may vary across the retained clinically sampled anatomical region pairs, including frontal, mesial temporal, and insular combinations. However, because electrode coverage was clinically determined, uneven across participants, and the nominal regional analyses were not corrected across all region‐ and frequency‐specific comparisons, these findings should be interpreted descriptively rather than as confirmatory evidence for specific anatomical effects. Positive and negative regional estimates indicate that the direction of coupling between window‐specific icEEG FC deviations and temporally aligned fMRI connectivity may differ across sampled region pairs, but the biological significance of these effects remains uncertain. Future studies with systematic anatomical sampling and appropriate correction for regional multiple comparisons will be needed to determine whether these spatial patterns reflect reproducible biological effects.

### 4.4 Motion Robustness

Dynamic coupling estimates were preserved when analyses were restricted to low‐motion runs. Overlapping confidence intervals across motion strata and the absence of systematic attenuation suggest that the observed cross‐modal associations were unlikely to be driven by residual head motion artifacts. In addition, static icEEG–fMRI coupling remained positive and FDR‐significant across all frequency bands after including subject‐level mean motion as an additional covariate, further supporting the robustness of the static coupling findings.

### 4.5 Relation to Prior Simultaneous icEEG–fMRI Studies

Simultaneous icEEG–fMRI datasets remain rare, limiting direct comparison. Ridley et al. (2017) reported cross‐modal correlations between icEEG and BOLD connectivity, strongest in lower‐frequency bands and non‐epileptogenic cortex. Our results are broadly consistent with this finding, demonstrating robust static associations between electrophysiological and haemodynamic connectivity across frequency bands. The present study extends this prior work by separating average sliding‐window electrophysiological connectivity from window‐level deviations around that average, allowing static and dynamic cross‐modal associations to be examined within the same modeling framework.

Mostame et al. (2025) proposed that large‐scale connectome dynamics operate as a multiplex system across timescales, showing that spatially similar icEEG‐ and fMRI‐derived states recur asynchronously. Extending this framework, we quantified cross‐modal coupling at the level of individual electrode‐pair connections rather than global state recurrence. Our findings suggest that temporal asynchrony at the global state level does not preclude window‐level coupling at the sampled electrode‐pair connection level.

A key distinction of the present study is scale. Prior simultaneous icEEG–fMRI investigations were based on small cohorts, limiting population‐level inference. Leveraging 48 subjects and hierarchical mixed‐effects modeling, we provide a population‐level characterization of cross‐modal coupling across individuals and clinically sampled anatomical contexts. Restricting analyses to non‐epileptogenic electrodes further reduced the influence of local epileptogenic activity on the observed cross‐modal associations.

## 5 Limitations

Several limitations warrant consideration. First, participants had drug‐resistant epilepsy and were undergoing clinical monitoring. Although analyses were restricted to non‐epileptogenic electrodes, chronic disease, seizure history, and medication exposure may influence electrophysiological and hemodynamic FC estimates (Ridley et al. 2017; Bettus et al. 2011), limiting generalizability to healthy populations.

Second, electrode placement was clinically determined, resulting in heterogeneous and incomplete spatial sampling. While hierarchical modeling across a large cohort strengthens inference, the analysis was limited to brain regions sampled by clinically implanted electrodes, and spatial coverage varied across participants.

Third, simultaneous acquisition poses technical challenges. Susceptibility artifacts near electrodes can produce local BOLD signal dropout, particularly in voxels containing the electrode contacts (Murta et al. 2015; Mercier et al. 2022; Aghakhani et al. 2015). To reduce reliance on a single dropout‐affected voxel, BOLD signals were extracted from a 3 × 3 × 3 voxel neighborhood centered on each bipolar midpoint, thereby incorporating surrounding voxels with less severe signal loss. Bipolar referencing reduces distant contamination, yet superficial artifacts, such as muscle activity, may persist (Jerbi et al. 2009).

Fourth, electrophysiological connectivity was quantified using BLP correlations (Schölvinck et al. 2013). Although this approach is well‐established for linking neural activity to BOLD fluctuations, alternative metrics, such as phase‐based or directed connectivity, may reveal complementary relationships (Bastos and Schoffelen 2016; Bakhshayesh et al. 2019). Relatedly, the present analyses focused on undirected FC; examining cross‐modal effective connectivity (Wei et al. 2020; Bönstrup et al. 2016; Vaudano et al. 2013; Murta et al. 2012) remains an important direction for future work. In addition, the high‐gamma analysis was restricted to 51–100 Hz and therefore did not capture broadband high‐gamma or higher‐frequency icEEG activity (Frauscher and Gotman 2019). Future work should directly examine broadband high‐gamma and higher‐frequency icEEG activity in relation to both regional BOLD amplitude and inter‐regional BOLD connectivity.

Fifth, the spatially stratified analysis was exploratory. Although these results suggested that the strength and direction of dynamic icEEG–fMRI coupling may vary across implanted anatomical regions, uneven clinical sampling and the absence of correction across all region‐ and frequency‐specific comparisons preclude strong anatomical inference from individual region‐pair effects.

Finally, the dynamic analyses used sliding‐window FC estimates, which may introduce temporal dependence between adjacent windows, particularly when windows overlap (Shakil 2016). Although the LMMs accounted for clustering by subject, run, sampled electrode‐pair connection, and subject‐level variability in the window‐specific ΔicEEG effect, they did not explicitly model autoregressive residual structure across sequential windows. ICC analyses were useful for characterizing between‐subject variance, but they cannot determine whether within‐subject window‐level variance reflects independent fluctuations, random noise, or serially autocorrelated structure. Thus, ICC values should not be interpreted as evidence that residual temporal autocorrelation was absent. However, when analyses were repeated using non‐overlapping 120 s windows, ΔicEEG effects remained positive across all frequency bands, although they did not survive FDR correction. This suggests that the direction of the dynamic coupling effects was not driven solely by overlap‐induced dependence between adjacent windows, while also indicating that statistical significance was weaker when window overlap was removed.

## 6 Conclusions

Resting‐state fMRI connectivity was associated with both average electrophysiological coupling and window‐level fluctuations in icEEG connectivity across sampled electrode‐pair connections. Static icEEG connectivity was robustly associated with corresponding BOLD FC across frequency bands, whereas dynamic fMRI connectivity showed significant band‐wise associations with window‐specific icEEG FC deviations, with the strongest primary evidence in the alpha, beta, and low‐gamma bands. Together, these findings provide population‐level evidence from simultaneous icEEG–fMRI that haemodynamic FC relates to both average electrophysiological coupling across sampled connections and window‐level fluctuations in electrophysiological connectivity.

## Author Contributions

**Tahereh Rashnavadi:** conceptualization, formal analysis, writing – original draft, review and editing, visualization. **Kristina Sabaroedin:** conceptualization, data curation, writing – review and editing. **William Wilson:** data curation. **Daniel J. Pittman:** data curation. **Paolo Federico:** data curation, writing – review and editing. **Pierre Levan:** supervision, conceptualization, funding acquisition, writing – review and editing.

## Funding

This work was supported by the Natural Sciences and Engineering Research Council of Canada (NSERC; RGPIN‐2021‐02797), the Canadian Institutes of Health Research (CIHR; PJT‐183825), the Alberta Children’s Hospital Research Institute, and the Hotchkiss Brain Institute, University of Calgary. Dr. Rashnavadi was supported by the HBI Inspires Postdoctoral Fellowship in Neuroscience and the 2026 Canadian Neuroanalytics Scholars (CNS) Program.

## Conflicts of Interest

The authors declare no conflicts of interest.

## Supporting information


**Table S1:** Static icEEG–fMRI coupling estimates across frequency bands. Standardized fixed‐effect estimates $\left(\beta \right)$ and 95% confidence intervals are shown for the association between mean icEEG FC and mean fMRI FC across sampled electrode‐pair connections. Estimates are reported separately for full‐recording static FC and window‐averaged static FC. All effects survived Benjamini–Hochberg false discovery rate correction across frequency bands $\left(p_{\mathrm{FDR}}<0.001\right)$.
**Table S2:** Sensitivity analysis without fMRI spatial smoothing. Linear mixed‐effects model estimates are shown for the primary 6‐mm FWHM fMRI preprocessing pipeline and the no‐spatial‐smoothing fMRI pipeline across frequency bands. Static models were fit as: fMRI FC ~ icEEG FC + inter‐electrode distance + age + sex + random effects for subject and electrode pair; dynamic models were fit as: dynamic fMRI FC ~ mean icEEG FC + icEEG FC variability + inter‐electrode distance + age + sex + random effects for subject and electrode pair. The no‐smoothing analysis reproduced the direction and FDR‐corrected significance pattern of the main icEEG‐fMRI coupling effects, including true static, window‐averaged, and dynamic mean icEEG FC terms. The secondary icEEG FC variability term showed some smoothing‐dependent differences.
**Table S3:** Temporal‐shift sensitivity analysis for dynamic icEEG‐fMRI coupling. Linear mixed‐effects model estimates are shown for the primary 6‐mm FWHM fMRI preprocessing pipeline and the no‐spatial‐smoothing fMRI pipeline across frequency bands. Static models were fit as: fMRI FC ~ icEEG FC + inter‐electrode distance + age + sex + random effects for subject and electrode pair. Dynamic models were fit as: dynamic fMRI FC ~ mean icEEG FC + icEEG FC variability + inter‐electrode distance + age + sex + random effects for subject and electrode pair. The no‐smoothing analysis reproduced the direction and FDR‐corrected significance pattern of the main icEEG‐fMRI coupling effects, including true static, window‐averaged, and dynamic mean icEEG FC terms. The secondary icEEG FC variability term showed some smoothing‐dependent differences.
**Table S4:** Window‐parameter sensitivity analysis for dynamic icEEG‐fMRI coupling. Linear mixed‐effects model estimates are shown for dynamic icEEG‐fMRI coupling models repeated using 90 s windows with 45 s overlap, 120 s windows with 60 s overlap, 150 s windows with 75 s overlap, and non‐overlapping 120 s windows. For each condition, both icEEG FC and fMRI FC were recomputed using the corresponding window length and overlap. Models were fit as: dynamic fMRI FC ~ mean icEEG FC + icEEG FC variability + inter‐electrode distance + age + sex + random effects for subject and electrode pair. The 120 s/60 s condition was used in the primary analysis. The table reports the mean icEEG FC term and the icEEG FC variability term across frequency bands. The mean icEEG FC term remained positive and FDR‐significant across all window settings, while the icEEG FC variability term showed a consistent significance pattern, with significance only in the high‐gamma band.
**Table S5:** Mismatched‐edge negative‐control analysis. Linear mixed‐effects model estimates are shown after disrupting the anatomical correspondence between icEEG and fMRI edges within each subject and frequency band. For static analyses, fMRI FC values were reassigned to non‐corresponding icEEG edges within each subject and frequency band. For the dynamic analysis, window‐level fMRI FC values were reassigned to non‐corresponding icEEG edges within subject, run, window, and frequency band. Models were then refit using the same covariates and random‐effects structure as the primary analyses. Static mismatched‐edge associations were near zero and did not survive FDR correction in any frequency band. Dynamic mismatched‐edge effects were strongly attenuated and did not reproduce the primary matched‐edge alpha/beta pattern, indicating that the reported icEEG–fMRI coupling depended on anatomically corresponding electrode‐pair connections. n.s. indicates that the matched‐edge primary effect was not significant after FDR correction.
**Table S6:** Data coverage for each anatomical region pair included in the spatially stratified icEEG–fMRI coupling analysis. Tables report the number of window‐level observations, contributing subjects, and unique electrode‐pair connections for each region–region combination across the six anatomical regions (LF, LMT, LI, RF, RMT, RI). Only region pairs meeting predefined inclusion criteria (≥ 6 subjects, ≥ 5 unique electrode pairs, and ≥ 500 window‐level observations) were included in the statistical analyses shown in Figure 8. Abbreviations: LF, left frontal; LMT, left mesial temporal; LI, left insula; RF, right frontal; RMT, right mesial temporal; RI, right insula.
**Table S7:** Motion‐adjusted sensitivity analysis for static icEEG–fMRI coupling. Linear mixed‐effects model estimates are shown for full‐recording static FC and window‐averaged static FC models after adding subject‐level mean motion as an additional covariate. Models also included icEEG FC variability, inter‐electrode distance, age, sex, and random effects for subject and electrode pair. Coefficients represent standardized fixed‐effect estimates for the relevant icEEG FC term. All models included 6819 observations from 48 subjects. The main icEEG–fMRI coupling effects remained positive and FDR‐significant across all frequency bands for both static FC estimators.

## Data Availability

The data supporting this study are not publicly available due to patient privacy and ethical restrictions. All analysis code required to reproduce the results and figures is publicly available at: https://github.com/rashnavadi/Static‐and‐dynamic‐resting‐state‐fMRI‐icEEG‐FC.
