# Supplementary material for: Static and Dynamic Resting‐State fMRI Connectivity Show Frequency‐Resolved Associations With Intracranial EEG Connectivity
Source: Hum Brain Mapp. 2026 Sep 30;47(14):e70646. doi: 10.1002/hbm.70646 (PMC13625044; doi:10.1002/hbm.70646)
Supplement: Supplementary file 1 [file HBM-47-e70646-s001.docx]

**Title:** Static and dynamic resting-state fMRI connectivity exhibit frequency-dependent coupling to intracranial EEG

**Authors:** Tahereh Rashnavadi^1,2^, Kristina Sabaroedin^1,2,3^, William Wilson^2,4^, Daniel J Pittman^2,4^, Paolo Federico^1,2,4^, Pierre Levan^1,2,5,6^

1. Department of Radiology, University of Calgary, Canada
2. Hotchkiss Brain Institute, University of Calgary, Canada
3. Department of Paediatrics, University of Calgary, Canada
4. Department of Clinical Neurosciences, University of Calgary, Canada
5. Seaman Family MR Research Centre, University of Calgary, Canada
6. Alberta Children’s Hospital Research Institute, University of Calgary, Canada

**Corresponding author:**

Tahereh Rashnavadi

[rashnavadi.tahereh@ucalgary.ca](mailto:rashnavadi.tahereh@ucalgary.ca)

University of Calgary

3300 Hospital Drive NW

Calgary, AB, T2N 4N1

Canada

**Supplementary Materials**

**Materials and Methods**

**Intraclass correlation analysis**

To characterize the variance structure of windowed functional connectivity (FC) across modalities, intraclass correlation coefficients (ICC) were computed separately for fMRI and for each icEEG frequency band. ICC analyses were used descriptively to estimate the proportion of variance attributable to between-subject differences relative to residual within-subject and edge-level variation in the windowed FC data.

ICCs were estimated using unconditional random-intercept linear mixed models (LMMs) fitted separately for each modality and each icEEG frequency band. For fMRI, models were applied to Fisher z–transformed windowed fMRI FC values. For icEEG, models were applied to band-limited windowed icEEG FC values in the delta, theta, alpha, beta, low-gamma, and high-gamma bands. Data were pooled across electrode-pair connections, time windows, and runs.

The ICCs were estimated from models of the form:

$$\mathrm{FC}_{s,e,w}=\beta_{0}+u_{s}+\varepsilon_{s,e,w},$$

where $\mathrm{FC}_{edge,win}$ denotes window-level dynamic connectivity for a given connection (or edge) and time window. ICC was defined as:

$$\mathrm{ICC}=\frac{\sigma_{\mathrm{subject}}^{2}}{\sigma_{\mathrm{subject}}^{2}+\sigma_{\varepsilon}^{2}}$$

Uncertainty in ICC estimates was quantified via subject-level bootstrap resampling with 500 iterations, with results reported as mean ± SD across bootstrap iterations. These ICC analyses provide a descriptive context for the variance structure of windowed FC across modalities and frequency bands. They were not used as inferential tests of band-specific icEEG–fMRI coupling and should not be interpreted as evidence that residual temporal autocorrelation was absent.

**Static icEEG–fMRI coupling analysis**

Linear mixed-effects models were fitted separately for each icEEG frequency band to test whether spatial variations in average icEEG FC across sampled electrode-pair connections was associated with corresponding variations in average fMRI FC. Separate models were fitted for two static FC estimators: full-recording static FC and window-averaged FC.

For the full-recording static FC model, the dependent variable was full-recording static fMRI FC, and the primary predictor was full-recording static icEEG FC for the corresponding electrode-pair connection and frequency band. For the window-averaged FC model, the dependent variable was window-averaged fMRI FC, and the primary predictor was window-averaged icEEG FC for the corresponding electrode-pair connection and frequency band.

All static models included icEEG FC variability, defined as the standard deviation of windowed icEEG FC across retained windows and runs for each subject, electrode-pair connection, and frequency band. Inter-electrode distance was included as a covariate to account for systematic distance-related effects on connectivity estimates. Distance was defined as the Euclidean distance between bipolar electrode-pair midpoints, log-transformed, and mean-centered. Age and sex were included as additional covariates. Random effects were included for subject and electrode-pair connection to account for repeated observations and inter-individual variability.

The full-recording static model was:

$${fMRI\_FC}_{s,e}^{\mathrm{full}}=\beta_{0}+\beta_{1}{icEEG\_FC}_{s,e}^{\mathrm{full}}+\beta_{2}SD({icEEG\_FC}_{s,e}^{\mathrm{win}})+\beta_{3}\mathrm{logDist}_{s,e}+\beta_{4}\mathrm{age}_{s}+\beta_{5}\mathrm{sex}_{s}+u_{s}+v_{e}+\epsilon_{s,e}$$

The window-averaged static model was:

$${fMRI\_FC}_{s,e}^{\mathrm{winAvg}}=\beta_{0}+\beta_{1}{icEEG\_FC}_{s,e}^{\mathrm{winAvg}}+\beta_{2}SD({icEEG\_FC}_{s,e}^{\mathrm{win}})+\beta_{3}\mathrm{logDist}_{s,e}+\beta_{4}\mathrm{age}_{s}+\beta_{5}\mathrm{sex}_{s}+u_{s}+v_{e}+\epsilon_{s,e}$$

Here, $s$ indexes subjects and $e$ indexes electrode-pair connections. The fixed effect of interest was $\beta_{1}$, which tested whether average icEEG FC was associated with corresponding average fMRI FC across sampled electrode-pair connections. Multiple comparisons across frequency bands were controlled using Benjamini–Hochberg false discovery rate correction.

**Dynamic window-level icEEG–fMRI coupling analysis**

Dynamic icEEG–fMRI coupling was tested using window-level LMMs fitted separately for each icEEG frequency band. The dependent variable was windowed fMRI FC for each subject, electrode-pair connection, run, and time window. As described in the main Methods, windowed icEEG FC was decomposed into a mean sliding-window icEEG FC term and a window-specific icEEG FC deviation term $\left( \Delta icEEG \right)$. Here, we provide the full model specification used for this analysis. The dynamic model was:

$${fMRI\_FC}_{s,e,r,w}^{\mathrm{win}}=\beta_{0}+\beta_{1}\overline{{icEEG\_FC}_{s,e}^{\mathrm{win}}}+\beta_{2}\Delta{icEEG\_FC}_{s,e,r,w}^{\mathrm{win}}+\beta_{3}SD({icEEG\_FC}_{s,e}^{\mathrm{win}})+\beta_{4}\mathrm{logDist}_{s,e}+\beta_{5}\mathrm{age}_{s}+\beta_{6}\mathrm{sex}_{s}+u_{s}+q_{s,r}+v_{e}+\epsilon_{s,e,r,w}$$

Here, $s$ indexes subjects, $e$ indexes electrode-pair connections, $r$ indexes runs, and $w$ indexes time windows. $\overline{{icEEG\_FC}_{s,e}^{\mathrm{win}}}$ represents the mean sliding-window icEEG FC across retained windows and runs for a given subject, electrode-pair connection, and frequency band. $\Delta{icEEG\_FC}_{s,e,r,w}^{\mathrm{win}}$ represents the window-specific deviation from that mean. $SD({icEEG\_FC}_{s,e}^{\mathrm{win}})$ represents the standard deviation of windowed icEEG FC across retained windows and runs for each subject, electrode-pair connection, and frequency band.

The fixed effect of interest was $\beta_{2}$, which tested whether window-specific icEEG FC deviations were associated with temporally aligned fluctuations in fMRI FC after accounting for mean sliding-window icEEG FC, overall icEEG FC variability, inter-electrode distance, age, and sex. Random effects were included for subject, run nested within subject, and electrode-pair connection to account for repeated observations across subjects, runs, windows, and edges.

To characterize inter-individual variability in dynamic coupling, additional models allowed subject-specific slopes for the $\Delta icEEG$ term. Subject-specific dynamic coupling estimates were derived as best linear unbiased predictors, defined as the sum of the fixed-effect coefficient and the corresponding subject-level random effect. These regularized subject-specific slopes were used to visualize inter-individual variability in dynamic icEEG–fMRI coupling across frequency bands.

**Table S1. Static icEEG–fMRI coupling estimates across frequency bands. Standardized fixed-effect estimates** $\left( \beta\right)$ **and 95% confidence intervals are shown for the association between mean icEEG FC and mean fMRI FC across sampled electrode-pair connections. Estimates are reported separately for full-recording static FC and window-averaged static FC. All effects survived Benjamini–Hochberg false discovery rate correction across frequency bands** $\left( p_{\mathrm{FDR}} < 0.001 \right)$**.**

| Frequency band | Full-recording static FC, β (95% CI) | Window-averaged static FC, β (95% CI) |
| --- | --- | --- |
| Delta | 0.77 [0.62, 0.93] | 0.76 [0.61, 0.92] |
| Theta | 0.87 [0.70, 1.05] | 0.91 [0.73, 1.10] |
| Apha | 1.12 [0.89, 1.34] | 1.16 [0.91, 1.40] |
| Beta | 1.51 [1.21, 1.80] | 1.80 [1.46, 2.14] |
| Low gamma | 1.69 [1.25, 2.14] | 2.36 [1.81, 2.90] |
| High gamma | 0.95 [0.66, 1.23] | 1.14 [0.83, 1.44] |

**Table S2. Sensitivity analysis without fMRI spatial smoothing.** Linear mixed-effects model estimates are shown for the primary 6-mm FWHM fMRI preprocessing pipeline and the no-spatial-smoothing fMRI pipeline across frequency bands. Static models were fit as: fMRI FC ~ icEEG FC + inter-electrode distance + age + sex + random effects for subject and electrode pair; dynamic models were fit as: dynamic fMRI FC ~ mean icEEG FC + icEEG FC variability + inter-electrode distance + age + sex + random effects for subject and electrode pair. The no-smoothing analysis reproduced the direction and FDR-corrected significance pattern of the main icEEG-fMRI coupling effects, including true static, window-averaged, and dynamic mean icEEG FC terms. The secondary icEEG FC variability term showed some smoothing-dependent differences.

| icEEG–fMRI Coupling | Band | Beta_6mm | CI_6mm | pFDR_6mm | Beta_no_smoothing | CI_no_smoothing | pFDR_no_smoothing | Consistent Across Spatial Smootings |
| --- | --- | --- | --- | --- | --- | --- | --- | --- |
| True Static | delta | 0.772 | [0.616, 0.928] | <0.001 | 0.491 | [0.376, 0.605] | <0.001 | Yes |
| True Static | theta | 0.874 | [0.701, 1.048] | <0.001 | 0.597 | [0.471, 0.723] | <0.001 | Yes |
| True Static | alpha | 1.117 | [0.888, 1.345] | <0.001 | 0.770 | [0.613, 0.926] | <0.001 | Yes |
| True Static | beta | 1.506 | [1.212, 1.800] | <0.001 | 1.108 | [0.907, 1.309] | <0.001 | Yes |
| True Static | Low gamma | 1.692 | [1.246, 2.139] | <0.001 | 1.232 | [0.909, 1.556] | <0.001 | Yes |
| True Static | high_gamma | 0.946 | [0.662, 1.230] | <0.001 | 0.656 | [0.477, 0.835] | <0.001 | Yes |
| Window-averaged | delta | 0.763 | [0.608, 0.918] | <0.001 | 0.499 | [0.381, 0.616] | <0.001 | Yes |
| Window-averaged | theta | 0.913 | [0.725, 1.101] | <0.001 | 0.594 | [0.458, 0.729] | <0.001 | Yes |
| Window-averaged | alpha | 1.158 | [0.915, 1.401] | <0.001 | 0.768 | [0.603, 0.933] | <0.001 | Yes |
| Window-averaged | beta | 1.800 | [1.461, 2.139] | <0.001 | 1.328 | [1.077, 1.578] | <0.001 | Yes |
| Window-averaged | low_gamma | 2.357 | [1.813, 2.901] | <0.001 | 1.761 | [1.364, 2.158] | <0.001 | Yes |
| Window-averaged | high_gamma | 1.135 | [0.826, 1.444] | <0.001 | 0.854 | [0.642, 1.065] | <0.001 | Yes |
| icEEG Dynamic FC: Mean | delta | 0.376 | [0.316, 0.436] | <0.001 | 0.264 | [0.214, 0.314] | <0.001 | Yes |
| icEEG Dynamic FC: Mean | theta | 0.624 | [0.550, 0.699] | <0.001 | 0.425 | [0.363, 0.488] | <0.001 | Yes |
| icEEG Dynamic FC: Mean | alpha | 0.643 | [0.560, 0.727] | <0.001 | 0.470 | [0.401, 0.539] | <0.001 | Yes |
| icEEG Dynamic FC: Mean | beta | 0.900 | [0.793, 1.006] | <0.001 | 0.756 | [0.668, 0.845] | <0.001 | Yes |
| icEEG Dynamic FC: Mean | low_gamma | 0.817 | [0.678, 0.956] | <0.001 | 0.787 | [0.672, 0.902] | <0.001 | Yes |
| icEEG Dynamic FC: Mean | high_gamma | 0.599 | [0.476, 0.722] | <0.001 | 0.589 | [0.486, 0.691] | <0.001 | Yes |
| icEEG Dynamic FC: Variability | delta | 0.072 | [-0.140, 0.285] | 0.505 | 0.079 | [-0.097, 0.256] | 0.456 | Yes |
| icEEG Dynamic FC: Variability | theta | -0.119 | [-0.331, 0.093] | 0.443 | -0.105 | [-0.282, 0.071] | 0.363 | Yes |
| icEEG Dynamic FC: Variability | alpha | 0.238 | [-0.041, 0.518] | 0.284 | 0.260 | [0.028, 0.493] | 0.084 | Yes |
| icEEG Dynamic FC: Variability | beta | 0.161 | [-0.177, 0.499] | 0.443 | -0.287 | [-0.568, -0.007] | 0.090 | Non-significant in both analyses |
| icEEG Dynamic FC: Variability | Low gamma | 0.156 | [-0.184, 0.495] | 0.443 | -0.445 | [-0.725, -0.164] | 0.011 | Different significance pattern |
| icEEG Dynamic FC: Variability | High gamma | 0.626 | [0.317, 0.935] | <0.001 | 0.025 | [-0.231, 0.280] | 0.850 | Different significance pattern |

**Table S3. Temporal-shift sensitivity analysis for dynamic icEEG-fMRI coupling.** Linear mixed-effects model estimates are shown for the primary 6-mm FWHM fMRI preprocessing pipeline and the no-spatial-smoothing fMRI pipeline across frequency bands. Static models were fit as: fMRI FC ~ icEEG FC + inter-electrode distance + age + sex + random effects for subject and electrode pair. Dynamic models were fit as: dynamic fMRI FC ~ mean icEEG FC + icEEG FC variability + inter-electrode distance + age + sex + random effects for subject and electrode pair. The no-smoothing analysis reproduced the direction and FDR-corrected significance pattern of the main icEEG-fMRI coupling effects, including true static, window-averaged, and dynamic mean icEEG FC terms. The secondary icEEG FC variability term showed some smoothing-dependent differences.

| icEEG Dynamic FC | Band | Beta_lag0s | pFDR_lag0s | Beta_lag3s | pFDR_lag3s | Beta_lag6s | pFDR_lag6s | Beta_lag9s | pFDR_lag9s | Consistent Across Lags |
| --- | --- | --- | --- | --- | --- | --- | --- | --- | --- | --- |
| Mean | delta | 0.380 | <0.001 | 0.377 | <0.001 | 0.376 | <0.001 | 0.376 | <0.001 | Yes |
| Mean | theta | 0.644 | <0.001 | 0.625 | <0.001 | 0.624 | <0.001 | 0.624 | <0.001 | Yes |
| Mean | alpha | 0.685 | <0.001 | 0.645 | <0.001 | 0.643 | <0.001 | 0.650 | <0.001 | Yes |
| Mean | beta | 0.916 | <0.001 | 0.903 | <0.001 | 0.900 | <0.001 | 0.906 | <0.001 | Yes |
| Mean | low_gamma | 0.816 | <0.001 | 0.820 | <0.001 | 0.817 | <0.001 | 0.819 | <0.001 | Yes |
| Mean | high_gamma | 0.628 | <0.001 | 0.599 | <0.001 | 0.599 | <0.001 | 0.596 | <0.001 | Yes |
| Variability | delta | 0.061 | 0.577 | 0.071 | 0.514 | 0.072 | 0.505 | 0.075 | 0.490 | Yes |
| Variability | theta | -0.154 | 0.460 | -0.117 | 0.474 | -0.119 | 0.443 | -0.147 | 0.370 | Yes |
| Variability | alpha | 0.098 | 0.577 | 0.237 | 0.289 | 0.238 | 0.284 | 0.188 | 0.370 | Yes |
| Variability | beta | 0.138 | 0.551 | 0.158 | 0.474 | 0.161 | 0.443 | 0.119 | 0.490 | Yes |
| Variability | low_gamma | 0.163 | 0.539 | 0.147 | 0.474 | 0.156 | 0.443 | 0.129 | 0.490 | Yes |
| Variability | high_gamma | 0.434 | 0.002 | 0.621 | <0.001 | 0.626 | <0.001 | 0.605 | <0.001 | Yes |

**Table S4. Window-parameter sensitivity analysis for dynamic icEEG-fMRI coupling.** Linear mixed-effects model estimates are shown for dynamic icEEG-fMRI coupling models repeated using 90 s windows with 45 s overlap, 120 s windows with 60 s overlap, 150 s windows with 75 s overlap, and non-overlapping 120 s windows. For each condition, both icEEG FC and fMRI FC were recomputed using the corresponding window length and overlap. Models were fit as: dynamic fMRI FC ~ mean icEEG FC + icEEG FC variability + inter-electrode distance + age + sex + random effects for subject and electrode pair. The 120 s/60 s condition was used in the primary analysis. The table reports the mean icEEG FC term and the icEEG FC variability term across frequency bands. The mean icEEG FC term remained positive and FDR-significant across all window settings, while the icEEG FC variability term showed a consistent significance pattern, with significance only in the high-gamma band.

| icEEG Dynamic FC | Band | Beta_90s_45s | pFDR_90s_45s | Beta_120s_60s_primary | pFDR_120s_60s_primary | Beta_150s_75s | pFDR_150s_75s | Beta_120s_0s_nonoverlap | pFDR_120s_0s_nonoverlap | Consistent Across Windows |
| --- | --- | --- | --- | --- | --- | --- | --- | --- | --- | --- |
| Mean | delta | 0.388 | <0.001 | 0.376 | <0.001 | 0.368 | <0.001 | 0.368 | <0.001 | Yes |
| Mean | theta | 0.654 | <0.001 | 0.624 | <0.001 | 0.621 | <0.001 | 0.632 | <0.001 | Yes |
| Mean | alpha | 0.664 | <0.001 | 0.643 | <0.001 | 0.651 | <0.001 | 0.667 | <0.001 | Yes |
| Mean | beta | 0.912 | <0.001 | 0.900 | <0.001 | 0.897 | <0.001 | 0.919 | <0.001 | Yes |
| Mean | low_gamma | 0.828 | <0.001 | 0.817 | <0.001 | 0.797 | <0.001 | 0.858 | <0.001 | Yes |
| Mean | high_gamma | 0.602 | <0.001 | 0.599 | <0.001 | 0.599 | <0.001 | 0.613 | <0.001 | Yes |
| Variability | delta | 0.015 | 0.890 | 0.072 | 0.505 | 0.075 | 0.472 | 0.083 | 0.573 | Yes |
| Variability | theta | -0.176 | 0.270 | -0.119 | 0.443 | -0.118 | 0.472 | -0.127 | 0.374 | Yes |
| Variability | alpha | 0.215 | 0.270 | 0.238 | 0.284 | 0.265 | 0.155 | 0.169 | 0.374 | Yes |
| Variability | beta | 0.168 | 0.414 | 0.161 | 0.443 | 0.139 | 0.472 | 0.084 | 0.729 | Yes |
| Variability | low_gamma | 0.163 | 0.414 | 0.156 | 0.443 | 0.138 | 0.472 | -0.010 | 0.948 | Yes |
| Variability | high_gamma | 0.649 | <0.001 | 0.626 | <0.001 | 0.606 | <0.001 | 0.500 | 0.003 | Yes |

**Table S5. Mismatched-edge negative-control analysis.** Linear mixed-effects model estimates are shown after disrupting the anatomical correspondence between icEEG and fMRI edges within each subject and frequency band. For static analyses, fMRI FC values were reassigned to non-corresponding icEEG edges within each subject and frequency band. For the dynamic analysis, window-level fMRI FC values were reassigned to non-corresponding icEEG edges within subject, run, window, and frequency band. Models were then refit using the same covariates and random-effects structure as the primary analyses. Static mismatched-edge associations were near zero and did not survive FDR correction in any frequency band. Dynamic mismatched-edge effects were strongly attenuated and did not reproduce the primary matched-edge alpha/beta pattern, indicating that the reported icEEG–fMRI coupling depended on anatomically corresponding electrode-pair connections. n.s. indicates that the matched-edge primary effect was not significant after FDR correction.

| Analysis | Frequency band | Matched beta | Matched pFDR | Mismatched beta | Mismatched Standar Error | Mismatched t | Mismatched p_FDR_ | Interpretation |
| --- | --- | --- | --- | --- | --- | --- | --- | --- |
| Full-recording static FC | Delta | 0.772 | <0.001 | -0.008 | 0.016 | -0.54 | 0.670 | Near-zero after mismatch |
| Full-recording static FC | Theta | 0.874 | <0.001 | -0.009 | 0.015 | -0.55 | 0.670 | Near-zero after mismatch |
| Full-recording static FC | Alpha | 1.117 | <0.001 | 0.006 | 0.015 | 0.43 | 0.670 | Near-zero after mismatch |
| Full-recording static FC | Beta | 1.506 | <0.001 | 0.022 | 0.016 | 1.44 | 0.449 | Near-zero after mismatch |
| Full-recording static FC | Low gamma | 1.692 | <0.001 | -0.012 | 0.017 | -0.69 | 0.670 | Near-zero after mismatch |
| Full-recording static FC | High gamma | 0.946 | <0.001 | -0.024 | 0.015 | -1.62 | 0.449 | Near-zero after mismatch |
| Window-averaged static FC | Delta | 0.763 | <0.001 | -0.006 | 0.015 | -0.41 | 0.828 | Near-zero after mismatch |
| Window-averaged static FC | Theta | 0.913 | <0.001 | -0.012 | 0.014 | -0.83 | 0.815 | Near-zero after mismatch |
| Window-averaged static FC | Alpha | 1.158 | <0.001 | 0.002 | 0.015 | 0.11 | 0.911 | Near-zero after mismatch |
| Window-averaged static FC | Beta | 1.800 | <0.001 | 0.020 | 0.015 | 1.32 | 0.561 | Near-zero after mismatch |
| Window-averaged static FC | Low gamma | 2.357 | <0.001 | -0.007 | 0.017 | -0.40 | 0.828 | Near-zero after mismatch |
| Window-averaged static FC | High gamma | 1.135 | <0.001 | -0.019 | 0.014 | -1.35 | 0.561 | Near-zero after mismatch |
| Dynamic FC: window-specific icEEG FC deviation term | Delta |  | n.s. | 0.006 | 0.002 | 3.07 | 0.013 | Strongly attenuated; primary pattern not reproduced |
| Dynamic FC: window-specific icEEG FC deviation term | Theta |  | n.s. | 0.005 | 0.002 | 2.29 | 0.044 | Strongly attenuated; primary pattern not reproduced |
| Dynamic FC: window-specific icEEG FC deviation term | Alpha | 0.084 | 0.009 | 0.006 | 0.002 | 2.70 | 0.021 | Strongly attenuated; primary pattern not reproduced |
| Dynamic FC: window-specific icEEG FC deviation term | Beta | 0.157 | 0.009 | 0.001 | 0.002 | 0.37 | 0.712 | Strongly attenuated; primary pattern not reproduced |
| Dynamic FC: window-specific icEEG FC deviation term | Low gamma |  | n.s. | -0.004 | 0.002 | -1.89 | 0.089 | Strongly attenuated; primary pattern not reproduced |
| Dynamic FC: window-specific icEEG FC deviation term | High gamma |  | n.s. | -0.002 | 0.002 | -0.64 | 0.626 | Strongly attenuated; primary pattern not reproduced |

**Table S6.** **Data coverage for each anatomical region pair included in the spatially stratified icEEG–fMRI coupling analysis.** Tables report the number of window-level observations, contributing subjects, and unique electrode-pair connections for each region–region combination across the six anatomical regions (LF, LMT, LI, RF, RMT, RI). Only region pairs meeting predefined inclusion criteria (≥ 6 subjects, ≥ 5 unique electrode pairs, and ≥ 500 window-level observations) were included in the statistical analyses shown in **Fig. 8.** Abbreviations: LF, left frontal; LMT, left mesial temporal; LI, left insula; RF, right frontal; RMT, right mesial temporal; RI, right insula.

**Table S7. Motion-adjusted sensitivity analysis for static icEEG–fMRI coupling.** Linear mixed-effects model estimates are shown for full-recording static FC and window-averaged static FC models after adding subject-level mean motion as an additional covariate. Models also included icEEG FC variability, inter-electrode distance, age, sex, and random effects for subject and electrode pair. Coefficients represent standardized fixed-effect estimates for the relevant icEEG FC term. All models included 6819 observations from 48 subjects. The main icEEG–fMRI coupling effects remained positive and FDR-significant across all frequency bands for both static FC estimators.

| Static FC estimator | Frequency band | β | Standar Error | t | pFDR |
| --- | --- | --- | --- | --- | --- |
| Full-recording static FC | Delta | 0.191 | 0.015 | 12.37 | <0.001 |
| Full-recording static FC | Theta | 0.245 | 0.015 | 16.41 | <0.001 |
| Full-recording static FC | Alpha | 0.226 | 0.015 | 15.43 | <0.001 |
| Full-recording static FC | Beta | 0.244 | 0.015 | 16.22 | <0.001 |
| Full-recording static FC | Low gamma | 0.181 | 0.017 | 10.52 | <0.001 |
| Full-recording static FC | High gamma | 0.128 | 0.015 | 8.66 | <0.001 |
| Window-averaged static FC | Delta | 0.183 | 0.015 | 12.25 | <0.001 |
| Window-averaged static FC | Theta | 0.228 | 0.014 | 16.40 | <0.001 |
| Window-averaged static FC | Alpha | 0.215 | 0.014 | 15.15 | <0.001 |
| Window-averaged static FC | Beta | 0.244 | 0.015 | 16.52 | <0.001 |
| Window-averaged static FC | Low gamma | 0.187 | 0.016 | 11.55 | <0.001 |
| Window-averaged static FC | High gamma | 0.131 | 0.014 | 9.51 | <0.001 |
